# Expanding flavone and flavonol production capabilities in *Escherichia coli*

**DOI:** 10.3389/fbioe.2023.1275651

**Published:** 2023-10-18

**Authors:** Andrew Yiakoumetti, Erik K. R. Hanko, Yutong Zou, Jeremy Chua, Jakub Chromy, Ruth A. Stoney, Kris Niño G. Valdehuesa, Jack A. Connolly, Cunyu Yan, Katherine A. Hollywood, Eriko Takano, Rainer Breitling

**Affiliations:** Manchester Institute of Biotechnology, School of Chemistry, Faculty of Science and Engineering, University of Manchester, Manchester, United Kingdom

**Keywords:** flavonoids, flavones, flavonols, *Escherichia coli*, cytochrome P450, rapid prototyping, biotransformation

## Abstract

Flavones and flavonols are important classes of flavonoids with nutraceutical and pharmacological value, and their production by fermentation with recombinant microorganisms promises to be a scalable and economically favorable alternative to extraction from plant sources. Flavones and flavonols have been produced recombinantly in a number of microorganisms, with *Saccharomyces cerevisiae* typically being a preferred production host for these compounds due to higher yields and titers of precursor compounds, as well as generally improved ability to functionally express cytochrome P450 enzymes without requiring modification to improve their solubility. Recently, a rapid prototyping platform has been developed for high-value compounds in *E. coli*, and a number of gatekeeper (2*S*)-flavanones, from which flavones and flavonols can be derived, have been produced to high titers in *E. coli* using this platform. In this study, we extended these metabolic pathways using the previously reported platform to produce apigenin, chrysin, luteolin and kaempferol from the gatekeeper flavonoids naringenin, pinocembrin and eriodictyol by the expression of either type-I flavone synthases (FNS-I) or type-II flavone synthases (FNS-II) for flavone biosynthesis, and by the expression of flavanone 3-dioxygenases (F3H) and flavonol synthases (FLS) for the production of the flavonol kaempferol. In our best-performing strains, titers of apigenin and kaempferol reached 128 mg L^−1^ and 151 mg L^−1^ in 96-DeepWell plates in cultures supplemented with an additional 3 mM tyrosine, though titers for chrysin (6.8 mg L^−1^) from phenylalanine, and luteolin (5.0 mg L^−1^) from caffeic acid were considerably lower. In strains with upregulated tyrosine production, apigenin and kaempferol titers reached 80.2 mg L^−1^ and 42.4 mg L^−1^ respectively, without the further supplementation of tyrosine beyond the amount present in the rich medium. Notably, the highest apigenin, chrysin and luteolin titers were achieved with FNS-II enzymes, suggesting that cytochrome P450s can show competitive performance compared with non-cytochrome P450 enzymes in prokaryotes for the production of flavones.

## 1 Introduction

**FIGURE 1:**
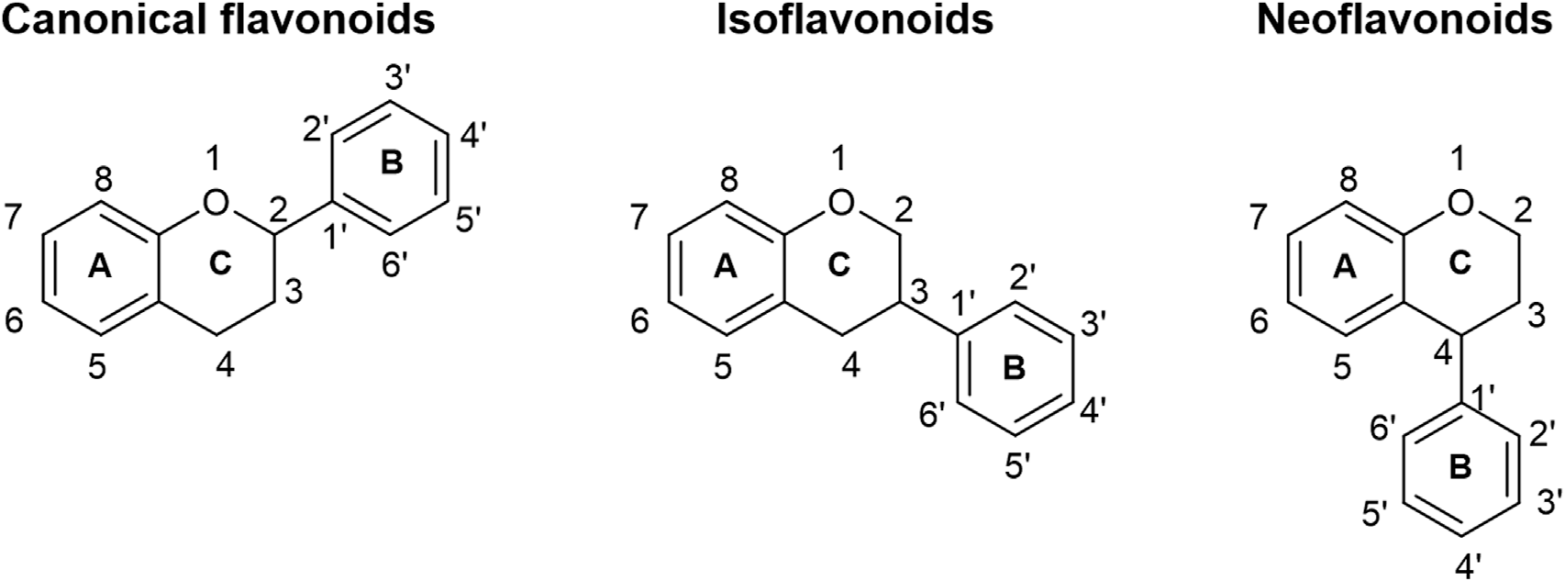
The basic skeletons of flavonoids. The B-ring may be connected to the C2 position of the heterocyclic C-ring (canonical flavonoids), the C3 position (isoflavonoids), or the C4 position (neoflavonoids).

Flavonoids constitute a diverse range of phenolic compounds that are predominantly found in plants and fungi. At least 8,000 different flavonoids have been identified across the plant and fungal kingdoms, all sharing a common phenylbenzopyran functionality where two aromatic rings (“A” and “B” rings) are linked through a heterocyclic C-ring (Marais et al., 2006) (Figure 1). Most members of the flavonoid family can be more precisely subclassified into one of at least six subgroups, including the (2*S*)-flavanones, flavones, isoflavones, flavonols, (2*S*)-flavanols, and anthocyanins, dependent on the degree of saturation of the heterocyclic C-ring, the position of the B-ring on the heterocyclic ring, as well as different substitution patterns across the basic skeletons of the flavonoid family (Sheng et al., 2020). Flavonoids confer a number of advantageous properties to plants and fungi, including pigmentation and aroma in order to help attract pollinators, as well as resistance to pathogens and protection against abiotic stress, such as reactive oxygen species and UV damage (Shirley, 1996). Flavonoids have also drawn interest for pharmaceutical and nutraceutical applications due to their myriad of predicted health benefits (Ballard and Maróstica, 2019), including antioxidant (Pietta, 2000), anti-cancer (Kopustinskiene et al., 2020), anti-inflammatory (Ginwala et al., 2019), cardioprotective (Testai et al., 2013), neuroprotective (Vauzour et al., 2008), antidiabetic (Al-Ishaq et al., 2019) and anti-anxiety (Nakazawa et al., 2003; Salehi et al., 2019) properties. Consequently, flavonoids have been targeted for commercial production, and this has traditionally relied on purification from plant sources or *de novo* chemical synthesis. However, low concentrations of flavonoids in plants lead to low yields after extraction (Trantas et al., 2015), while chemical synthesis of these complex compounds is expensive and unsustainable (Park et al., 2009). By contrast, biosynthesis of flavonoids in engineered microorganisms is seen as a scalable and reliable option, and a number of research groups have made progress in engineering a range of microorganisms, most notably *Saccharomyces cerevisiae* and *Escherichia coli*, for the production of a plethora of flavonoids (Sheng et al., 2020).

The use of *S. cerevisiae* has traditionally been a preferred option for flavonoid production, in part due to increased ease of expression of cytochrome P450 enzymes (Gonzalez and Korzekwa, 1995. Toward increasing titers of flavonoid production by *E. coli* for commercial exploitation, Dunstan et al. used a previously described rapid prototyping pipeline (Robinson et al., 2020) to develop metabolic routes with improved flux to naringenin, pinocembrin, eriodictyol and homoeriodictyol (Supplementary Figure S1) (Dunstan et al., 2020)—these compounds are all (2*S*)-flavanones and are considered to be gatekeeper flavonoids from which other flavonoids with useful properties can be derived (Dunstan et al., 2020). Notably, naringenin was produced to a titer of 466 mg L^−1^ in rich medium supplemented with tyrosine (3 mM) and pinocembrin was produced to a titer of 198 mg L^−1^ in rich medium supplemented with phenylalanine (3 mM). Similarly, eriodictyol was produced to a titer of 88 mg L^−1^ in rich medium supplemented with caffeic acid (3 mM).

To reduce the need to supplement tyrosine for the production of naringenin, a plasmid (pTyr+) overexpressing phosphoenolpyruvate synthase (encoded by *ppsA*) as well as feedback-resistant variants of 3-deoxy-7-phosphoheptulonate synthase (encoded by *aroG*) and bifunctional chorismite mutase/prephenate dehydrogenase (encoded by *tyrA*) was used, and this enabled naringenin titers of 396 mg L^−1^ in rich medium without further supplementation of tyrosine (Dunstan et al., 2020). In a Δ*tyrR* Δ*pheLA* Double KnockOut (DKO) strain (Robinson et al., 2020)—to derepress aromatic amino acid formation and restrict carbon flux to phenylalanine—these titers increased to 484 mg L^−1^ (Dunstan et al., 2020). To ensure that enough malonyl-CoA was present for assimilation into flavonoid biosynthesis, cerulenin was added (Dunstan et al., 2020), as this inhibits fatty acid biosynthesis and increases the cellular availability of malonyl-CoA (Santos et al., 2011).

In this study, we sought to extend the metabolic pathways from the gatekeeper flavonoids to chrysin, luteolin, kaempferol and apigenin. Apigenin shows anti-inflammatory (Elsisi et al., 2005), antidepressant (Nakazawa et al., 2003), neuroprotective (Zhao et al., 2013) and anti-cancer (Fang et al., 2005) properties, while kaempferol has shown anti-cancer (Chen and Chen, 2013) and cardioprotective properties (Feng et al., 2021). Similarly, luteolin has shown cardioprotective (Luo et al., 2017) and neuroprotective (Kempuraj et al., 2021) properties, and chrysin is reportedly an aromatase inhibitor (Balam et al., 2020). While these compounds have previously been produced in microorganisms (Table 1), we sought to harness the flavonoid gatekeeper platform to screen alternative enzymes for the production of these compounds, and to boost titers.

**TABLE 1:** Summary of flavone and flavonol production reported in the literature.

Product	Titer [mg L^−1^]	Used biosynthesis enzymes	Organism; substrate	Malonyl-CoA upregulation	Reference
Kaempferol	27	AmF3H and AtFLS in naringenin chassis	*S. cerevisiae*; glucose	N/A	Rodriguez et al. (2017)
	347	AtF3H and AtFLS in naringenin chassis	*S. cerevisiae*; glucose	Co-expression of mutant ACC Shi et al. (2014)	Tartik et al. (2023)
	15.1	RmPAL, ScCCL, GeCHS, PlCHI, CsF3H, CuFLS	*E. coli*; tyrosine	Co-expression of CgACC Miyahisa et al. (2005)	Miyahisa et al. (2006)
Chrysin	9.4	RmPAL, ScCCL, GeCHS, PlCHI, PcFNS-I	*E. coli*; phenylalanine	Co-expression of CgACC Miyahisa et al. (2005)	Miyahisa et al. (2006)
Apigenin	13	RmPAL, ScCCL, GeCHS, PlCHI, PcFNS-I	*E. coli*; tyrosine	Co-expression of CgACC Miyahisa et al. (2005)	Miyahisa et al. (2006)
	30	Os4CL, PeCHS, MtCHI, PcFNS-I	*E. coli*; *p*-coumaric acid	N/A	Lee et al. (2015)
	110	Pc4CL, PhCHS, MsCHI, PcFNS-I	*E. coli*; *p*-coumaric acid	Cerulenin	Leonard et al. (2008)
Luteolin	4	Pc4CL, PhCHS, MsCHI, PcFNS-I	*E. coli*; caffeic acid	Cerulenin	Leonard et al. (2008)

Enzyme abbreviations: ACC, Acetyl-CoA carboxylase; CCL/4CL, Coumarate-CoA ligase; CHI, Chalcone isomerase; CHS, Chalcone synthase; F3H, Flavanone 3-dioxygenase; FLS, Flavonol synthase; FNS-I, Flavone synthase I; PAL, Phenylalanine ammonia lyase. Organism abbreviations prefixed to enzyme abbreviations: Am, *Astragalus mongholicus*; At, *Arabidopsis thaliana*; Cg, *Corynebacterium glutamicum*; Cs, *Citrus sinensis*; Cu, *Citrus unshiu*; Ge, *Glycyrrhiza echinata*; Ms, *Medicago sativa*; Mt, *Medicago truncatula*; Os, *Oryza sativa*; Pc, *Petroselinum crispum*; Pe, *Populus euramericana* Guiner; Ph, *Petunia hybrida*; Pl, *Pueraria lobata*; Rm, *Rhodotorula mucilaginosa*; Sc, *Streptomyces coelicolor*.

To identify pathways and enzymes that could extend the gatekeepers pathways to apigenin, chrysin, luteolin and kaempferol, we used the computational tools RetroPath2.0 (Delépine et al., 2018) and Selenzyme (Carbonell et al., 2018), combined with manual literature searching and curation. Two different type I flavone synthase (FNS-I) enzymes and five type II flavone synthase (FNS-II) enzymes, as well as one cytochrome P450 reductase (CPR) for the functional catalytic activity of FNS-II, were selected to produce apigenin, chrysin and luteolin from naringenin, pinocembrin and eriodictyol, respectively. Similarly, four flavanone-3-dioxygenase (F3H) enzymes and four flavonol synthase (FLS) enzymes were selected for the two-step production of kaempferol from naringenin (via dihydrokaempferol). To produce kaempferol, we first performed *in vitro* screening to select one F3H enzyme (from *Glycine max*) and one FLS enzyme (from *Citrus unshiu*), and then assembled the genes encoding these enzymes together, into a combinatorial library of plasmids with different regulatory elements. Finally, we utilized a rapid prototyping procedure to screen strains for the production of apigenin and kaempferol in rich medium supplemented with tyrosine, the production of luteolin in rich medium supplemented with caffeic acid, and the production of chrysin in rich medium supplemented with phenylalanine. We also used this same rapid prototyping procedure to screen strains for the production of apigenin and kaempferol in rich medium that was not supplemented with tyrosine.

## 2 Materials and methods

### 2.1 Pathway selection

Pathway design was performed using the retrosynthesis software RetroPath2.0 (Delépine et al., 2018), supplemented with manual curation and literature research. This software generates reaction sequences connecting target compounds to precursor metabolites within the host. Apigenin, chrysin, luteolin and kaempferol were used as target compounds, to be linked to *E. coli* host metabolites. The maximum pathway length was limited to 12 reactions, to avoid unrealistically long solutions. Reactions are based on reaction rules, which were downloaded from the RetroRules website (Duigou et al., 2018). High levels of reaction novelty were enabled by setting the reaction diameters to a minimum of 2 and a maximum of 16. This allowed reactions to be included in the output, in which the atoms central to the suggested chemical transformation match known reactions, but variation within the surrounding structures is allowed. RetroPath2.0 was run using the KNIME data analytics platform version 3.3.1 (Bock, 2007).

### 2.2 Enzyme selection

Suitable candidates for enzymatic steps towards the biosynthesis of chrysin, apigenin, luteolin and kaempferol in *E. coli* were identified using both the automated enzyme selection tool Selenzyme (Carbonell et al., 2018) and a literature search. Reaction EC number-based queries (1.14.11.22 for FNS-I and FNS-II, 1.14.11.9 for F3H, and 1.14.11.23 for FLS reactions) were used for the Selenzyme search with the default settings and with only reaction similarity and UniProt protein evidence scores being considered, so that taxonomically distant enzymes were not penalized. During the literature search and final selection of enzymes, three main factors were considered, namely, evidence of successful expression in *E. coli* or *S. cerevisiae*, evidence of catalytic activity with the target compounds, and previous kinetic characterization.

### 2.3 Growth media

LB Miller (Formedium LMM0102) and LB Miller agar (Formedium LMM0202) were used for routine strain construction and propagation, with SOC medium (Formedium SOC0202) being used as outgrowth medium for plasmid transformations. Flavonoids production was performed in TBPY medium (Formedium Terrific Broth Phosphate Buffered TBP0102 supplemented with 0.4% w/v glycerol). All media except SOC outgrowth medium were supplemented with the appropriate antibiotics for plasmid selection and maintenance. Carbenicillin was added to 100 μg mL^−1^ for Amp^R^ plasmids, kanamycin was added to 50 μg mL^−1^ for Km^R^ plasmids, and chloramphenicol was added to 20 μg mL^−1^ for Cm^R^ plasmids.

### 2.4 Plasmid and strain construction

Strain *E. coli* NEB5α was purchased from New England Biolabs, and strain *E. coli* NEB5α Δ*tyrR* Δ*pheLA* was constructed previously (Robinson et al., 2020). Plasmids SBC005753, SBC006456, SBC006524, SBC006845 were also constructed previously (Dunstan et al., 2020), and are detailed in Supplementary Table S1. Before DNA parts synthesis, candidate enzymes were evaluated for signal peptide cleavage sites and transmembrane motifs and truncated accordingly to improve protein solubility (Dyrløv Bendtsen et al., 2004; Hallgren et al., 2022). Next, gene parts were designed using PartsGenie, with RBS translation initiation rates set to 20,000 (Swainston et al., 2018), and custom-synthesized and cloned into pBbE2c-based expression plasmids (Lee et al., 2011) by Twist Bioscience (Supplementary Table S2). For *in vitro* enzyme activity screening, these plasmids were transformed into *E. coli* NEB5α for overexpression of individual F3H and FLS enzymes. Plasmids that were constructed for *in vivo* assays confer resistance to ampicillin, were assembled by HiFi DNA Assembly and traditional sub-cloning, and are summarized in Figure 3A; Supplementary Table S3. Sequences for these plasmids can be found in the Supplementary Material as Genbank standard format DNA maps as well as via the SynBioChem ICE repository (https://ice.synbiochem.co.uk). For the production of apigenin from tyrosine, Group I plasmids SBC015646–SBC015653 and Group II plasmids SBC015673–SBC015677 were transformed into *E. coli* NEB5α harboring SBC006456. The same plasmids were transformed into *E. coli* NEB5α harboring SBC006524 for the production of chrysin from phenylalanine, and into *E. coli* NEB5α harboring SBC006845 for the production of luteolin from caffeic acid. Group III and IV plasmids SBC015883–SBC015894 were transformed into *E. coli* NEB5α harboring SBC006456 for the production of kaempferol from tyrosine, and into *E. coli* NEB5α Δ*tyrR* Δ*pheLA* harboring SBC005753 and SBC006456 for the production of kaempferol from central metabolism. For the production of apigenin from central metabolism, plasmids SBC015646, SBC015648, SBC015650, SBC015652 and Group II plasmids SBC015673–SBC015677 were transformed into *E. coli* NEB5α Δ*tyrR* Δ*pheLA* harboring SBC005753 and SBC006456.

### 2.5 *In vitro* enzyme activity screening

Whole-cell extracts of *E. coli* NEB5α expressing the individual enzymes were prepared as described previously (Robinson et al., 2020). Enzyme assays were performed in 96-well microtiter plates (clear and flat bottom, Corning) in a final volume of 100 µL. Each reaction contained 80 µL lysate (corresponding to 160 µg of total protein), 5 µL of 3 mM substrate, 5 µL of 6 mM 2-oxoglutarate, 5 µL of 75 mM ascorbate, and 5 µL of 3 mM FeSO_4_ freshly dissolved in 100 mM Tris-HCl (pH 8.0). Plates were sealed and incubated at 30°C and 850 rpm for 10 min. Samples were mixed with an equal volume of 10% methanol and diluted further 100x and 1,000x using 40% methanol for LC-MS/MS analysis.

### 2.6 Flavonoid production

For the production of flavonoids in rich media supplemented with both glycerol and a pathway precursor (tyrosine, phenylalanine or caffeic acid), freshly transformed single colonies were used to inoculate 10 mL overnight seed cultures in TBPY medium, supplemented with the appropriate antibiotics. Seed cultures were incubated at 37°C, 180 rpm for 12–16 h. Each seed culture was normalized, in triplicate, to OD_600_ = 1 by diluting seed culture into 500 μL fresh TBPY medium (with appropriate antibiotics) in a 96-DeepWell OD-normalization plate. Main cultures were then inoculated by a 1 in 20 dilution from the OD-normalization plate—into 1 mL fresh TBPY with relevant antibiotics or, for strains expressing FNS-II enzymes, into 1 mL TBPY with relevant antibiotics and 5-aminolevulinic acid (5-ALA, 0.1 mM)—to OD_600_ = 0.05 in a new 96-DeepWell plate. Immediately after inoculation, cultures were incubated at 30°C, 850 rpm in a plate-incubator with 80% humidity until OD_600_ reached 1.5–5 (typically after 6–9 h), before substrate (tyrosine, phenylalanine or caffeic acid) was added to a final concentration of 3 mM. Cultures were induced by the addition of IPTG (0.1 mM). All cultures were incubated for a further 24–30 h at 30°C, 850 rpm with 80% humidity before being quenched by the addition of an equivalent volume of 100% methanol, followed by overnight storage at −80°C. For the production of apigenin and kaempferol in TBPY medium using strains with upregulated tyrosine production (DKO-derivative strains) and without supplementation of additional tyrosine, the same procedure was followed, with the following modification to the seed cultures: Three individual colonies were picked as biological replicates, and each inoculated into separate 1 mL cultures of TBPY medium and the appropriate antibiotics, in a 96-DeepWell plate (seed-culture plate). Seed cultures were incubated at 30°C, 850 rpm with 80% humidity for 16 h, and OD normalization was performed once for each repeat in the seed-culture plate, rather than thrice for each 10 mL seed culture performed for the other experiments. In addition, main cultures were inoculated by a 1 in 10 dilution from the OD-normalization plate to OD_600_ = 0.1.

### 2.7 Analysis by LC-MS/MS

Quenched samples were diluted further in 40% methanol before analysis of compound formation was performed by LC-MS/MS. LC-MS/MS was performed using a Waters ACQUITY H-Class UPLC^®^ equipped with an ACQUITY UPLC^®^ BEH C18 1.7 μm, 2.1 mm × 50 mm column and coupled to a Waters Xevo^®^ TQ-S Mass Spectrometer. MS parameters and MRM transitions for all compounds analyzed in this study are detailed in Supplementary Table S4, while LC methods are detailed in Supplementary Table S5.

## 3 Results

### 3.1 Pathway design

**FIGURE 2:**
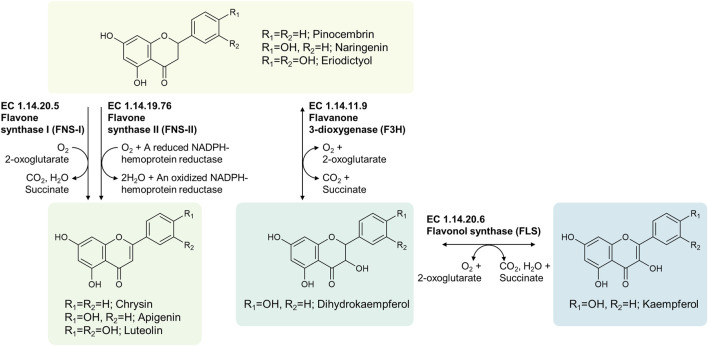
Biochemical routes to apigenin, chrysin, luteolin and kaempferol from 2(*S*)-flavanone gatekeepers.

RetroPath2.0 followed by manual curation of output files was used to identify classes of enzymes for the production of apigenin, chrysin and luteolin, and classes of enzymes which formed routes to kaempferol (Figure 2). Flavone synthase I (FNS-I, EC 1.14.20.5) enzymes and flavone synthase II (FNS-II, EC 1.14.19.76) enzymes both catalyze the oxidation of the (2*S*)-flavanones to their corresponding flavones, but they have different cofactor requirements. FNS-I enzymes oxidize the (2*S*)-flavanones with molecular oxygen, along with the concomitant conversion of 2-oxoglutarate to succinate and CO_2_, forming water as by-product of the reaction. By contrast, FNS-II enzymes catalyze the oxidation of (2*S*)-flavanones with molecular oxygen in a manner that simultaneously oxidizes a heme group within it, forming water as a byproduct by reducing molecular oxygen. Thus, both the substrate and the heme cofactor of the substrate are oxidized, and the liberated electrons are used to reduce molecular oxygen to water. To continue oxidizing the (2*S*)-flavanones, this heme group must be reduced once more, by the action of an NADPH-hemoprotein reductase, otherwise known as a cytochrome P450 reductase (CPR), using electrons from NADPH in order to do so. For the production of kaempferol, the top-ranked pathways suggested by RetroPath2.0 included the use of a flavanone 3-dioxygenase (F3H, EC 1.14.11.9) to form dihydrokaempferol from (2*S*)-naringenin and a flavonol synthase (FLS, EC 1.14.20.6) for the production of kaempferol from dihydrokaempferol.

### 3.2 Enzyme selection

By combining automated enzyme selection by Selenzyme with an independent literature search, two FNS-I and five FNS-II enzymes, as well as four F3H and four FLS enzymes, were selected to be assessed in this study (Table 2). All these enzymes met our selection criteria of having previously been expressed in *E. coli* or in *S. cerevisiae* and of having previously been shown to be catalytically active on some of our candidate substrates with *in vitro* or *in vivo* assays. Since FNS-II enzymes require CPRs for catalytic activity, a literature search was also performed to identify suitable CPR enzymes. CPR from *Arabidopsis thaliana* has been shown to be active and compatible with cytochrome P450 enzymes from a variety of plant sources (Li et al., 2019; Liu et al., 2023) and was therefore chosen here for co-expression with all the FNS-II enzymes.

**TABLE 2:** Enzymes selected for the production of apigenin, chrysin, luteolin, kaempferol and quercetin in this study.

Enzyme name	Uniprot accession number	Organism	Specific enzyme abbreviation	Reference	Identified using
Flavone synthase I (FNS-I, EC 1.14.20.5)	Q7XZQ8	*Petroselinum crispum*	PcFNS-I	Britsch (1990), Leonard et al. (2005), Miyahisa et al. (2006), Katsuyama et al. (2007)	Selenzyme
	A0A076U8J8	*Plagiochasma appendiculatum*	PaFNS-I	Han et al. (2014)	Literature
Flavone synthase II (FNS-II, EC 1.14.19.76)	A0A146AUI9	*Scutellaria baicalensis*	SbFNS-II	Zhao et al. (2016)	Literature
	M1L7D1	*Ocimum basilicum*	ObFNS-II	Berim and Gang (2013)	Selenzyme
	E9KBR8	*Glycine max*	GmFNS-II	Fliegmann et al. (2010)	Literature
	Q9XGT9	*Gerbera hybrida*	GhFNS-II	Martens and Forkmann (1999)	Literature
	A0A142FX91	*Lonicera japonica*	LjFNS-II	Wu et al. (2016)	Literature
Flavanone 3-dioxygenase (F3H, EC 1.14.11.9)	Q53B69	*Glycine max*	GmF3H	Ralston et al. (2005), Bong Gyu et al. (2008)	Literature
	Q7XM21	*Oryza sativa* ssp. *japonica*	OsF3H	Jeong Ho et al. (2008)	Literature
	Q9ZWR0	*Citrus sinensis*	CsF3H	Miyahisa et al. (2006), Owens et al. (2008)	Literature
	Q07353	*Petunia hybrida*	PhF3H	Britsch and Grisebach (1986), Britsch et al. (1992)	Selenzyme
Flavonol synthase (FLS, EC 1.14.20.6)	Q9ZWQ9	*Citrus unshiu*	CuFLS	Wellmann et al. (2002), Lukacin et al. (2003)	Selenzyme
	A2TJG2	*Camellia sinensis*	CsFLS	Lin et al. (2007)	Literature
	B4FS68	*Zea mays*	ZmFLS	Falcone Ferreyra et al. (2010)	Literature
	D0UZK7	*Ginkgo biloba*	GbFLS	Xu et al. (2012)	Selenzyme

### 3.3 *In vitro* assays

In order to narrow down the potential combinations of F3H and FLS enzymes for *in vivo* testing, an initial *in vitro* activity screening was conducted. While each enzyme had been previously purified and studied individually for substrate preference and catalytic activity (Britsch and Grisebach, 1986; Lukacin et al., 2003; Lin et al., 2007; Bong Gyu et al., 2008; Jeong Ho et al., 2008; Owens et al., 2008; Falcone Ferreyra et al., 2010; Xu et al., 2012), a comprehensive comparative evaluation under more physiologically relevant conditions using crude extracts was lacking. Therefore, biotransformation reactions were performed using whole-cell extracts of *E. coli* expressing the individual enzymes, and product formation was monitored using LC-MS/MS.

To assess the activity of F3H enzymes, naringenin was supplemented to whole-cell extracts containing these enzymes, and all four F3Hs successfully converted naringenin to dihydrokaempferol, with GmF3H demonstrating the best performance (Table 3). Likewise, to test the FLS enzymes, dihydrokaempferol was supplemented to whole-cell extracts containing these enzymes. However, since CuFLS and GbFLS are known to directly convert naringenin to kaempferol (Lukacin et al., 2003; Xu et al., 2012), all FLS enzymes were also tested with naringenin as a substrate.

**TABLE 3:** Whole-cell lysate activity screening of F3H and FLS enzyme candidates. Product yields (in %) obtained in *in vitro* enzyme assays. Reactions were performed using whole-cell lysates of *E. coli* expressing the individual enzymes, and the formation of products was monitored using LC-MS/MS. Reactions of 100 µL contained 160 µg of total protein, 150 µM substrate, 300 µM 2-oxoglutarate, 3.75 mM ascorbate, 150 µM iron(II) sulfate, 5 mM Tris-HCl (pH 8.0), and were incubated at 30°C and 850 rpm for 10 min. Yields were calculated from single biological replicates. n.d.–not detected. (−)–not tested. CsFLS and GbFLS showed no activity.

Enzyme	Naringenin→Dihydrokaempferol (%)	Naringenin→Kaempferol (%)	Dihydrokaempferol→Kaempferol (%)
GmF3H	13.5	n.d	–
OsF3H	4.7	n.d	–
CsF3H	3.3	n.d	–
PhF3H	0.4	n.d	–
CuFLS	7.7	2.8	59.9
ZmFLS	0.6	n.d	3.9

Among the four tested FLS enzymes, only CuFLS and ZmFLS showed product formation in whole-cell extracts. CuFLS exhibited a kaempferol yield of nearly 60% when supplemented with dihydrokaempferol. Due to CuFLS exhibiting a 15-fold higher activity towards dihydrokaempferol compared to ZmFLS, it was chosen for pathway assembly.

### 3.4 Pathway assembly and screening

**FIGURE 3:**
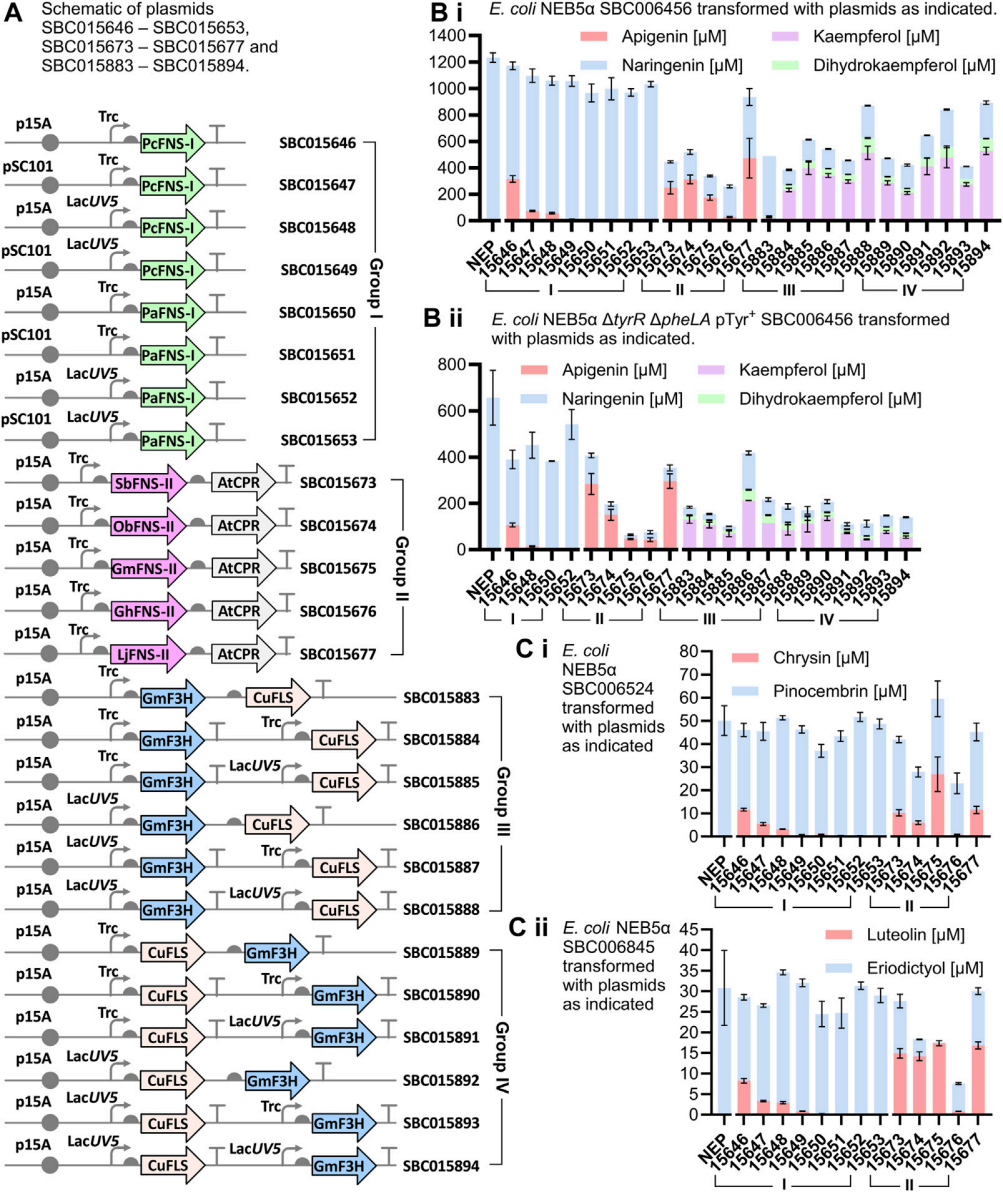
Production of flavonoids in whole-cell cultures and biotransformations. **(A)** Schematic figure of plasmids transformed into host strains for the conversion of gatekeeper flavonoids to flavones and flavonols. Gray circles: origins of replication; curved arrows: promoters; vertical lines capped by horizontal bars (T-shape): terminators; gray semi-circles: ribosome binding sites; green arrows: type I flavone synthase (FNS-I) genes; orchid arrows: type II flavone synthase (FNS-II) genes; grey arrows: cytochrome P450 reductase (CPR) genes; blue arrows: flavanone 3-dioxyngenase (F3H) genes; beige arrows: flavonol synthase (FLS) genes. Organism abbreviations prefixed to gene names: At, *Arabidopsis thaliana*; Cu, *Citrus unshiu*; Gh, *Gerbera hybrida*; Gm, *Glycine max*; Lj, *Lonicera japonica*; Ob, *Ocimum basilicum*; Pa, *Plagiochasma appendiculatum*; Pc, *Petroselinum crispum*; Sb, *Scutellaria baicalensis*. **(B)** i) Production of apigenin and kaempferol in *E. coli* NEB5α grown in TBPY medium supplemented with tyrosine (3 mM). **(B)** ii) Production of apigenin and kaempferol in *E. coli* NEB5α Δ*tyrR* Δ*pheLA* pTyr + grown in TBPY medium. **(C)** i) Production of chrysin in *E. coli* NEB5α grown in TBPY medium supplemented with phenylalanine (3 mM). **(C)** ii) Production of luteolin in *E. coli* NEB5α grown in TBPY medium supplemented with caffeic acid (3 mM). For graphs Bi, Bii, Ci and Cii, plasmid identifiers are prefixed by SBC0 (e.g., plasmid 15646 is plasmid SBC015646). NEP is a No Extra Plasmid negative control whereby the host strain is grown to produce only the (2*S*)-flavanone gatekeeper without expression of any flavone synthase, flavanone 3-monooxygenase or flavonol synthase enzymes. Error bars represent standard deviations of biological triplicates.

The production of apigenin and kaempferol from tyrosine (Figure 3B.i), chrysin from phenylalanine (Figure 3C.i), and luteolin from caffeic acid (Figure 3C.ii) was screened in separate experiments in 96-DeepWell plates. FNS-I from *Petroselinum crispum* (PcFNS-I) has previously been successfully used for the production of apigenin, chrysin and luteolin in *E. coli* (Table 1), and to optimize the production of these compounds when paired with the strains developed by Dunstan *et al.*, its expression was tested from both *trc* (strong) and *lacUV5* (weak) promoters, in p15A (medium copy) and pSC101 (low copy) plasmid backbones (Figure 3A, plasmids SBC015446–SBC015649). FNS-I from *Plagiochasma appendiculatum* (PaFNS-I) was tested in the same manner (plasmids SBC015650–SBC015653, Figure 3A), while FNS-II enzymes were all expressed from the *trc* promoter on p15A plasmid backbones (plasmids SBC015673–SBC015677). Genes encoding FNS-II enzymes were assembled into an operon with the gene encoding CPR from *A. thaliana* (Figure 3A, plasmids SBC015673–SBC015677); since it has been suggested that high expression levels of AtCPR increase the burden on the host cells (Li et al., 2019), the gene encoding AtCPR was implemented in an operon rather than being expressed from its own promoter. More specifically, it was encoded as the second gene in the operon rather than the first in the operon, which tends to be the highest expressed gene of an operon (Lim et al., 2011).

The highest titers reached from PcFNS-I were achieved when expressing its gene from a *trc* promoter on the p15A backbone, where apigenin titers reached 316 ± 25 μM (85.2 ± 6.8 mg L^−1^, Figure 3B.i). No apigenin (Figure 3B.i), chrysin (Figure 3C.i) or luteolin (Figure 3C.ii) production was observed when using PaFNS-I, while the highest titers of apigenin, 473 ± 150 μM (128 ± 41 mg L^−1^) were reached by the expression of FNS-II from *Lonicera japonica* (LjFNS-II). This is a similar titer to, and possibly a marginal improvement over, the 110 mg L^−1^ achieved by Leonard *et al.* (Leonard et al., 2008), and while the final cell density in our cultures might be higher (OD_600_ ≈ 20), these titers were achieved without the supplementation of cerulenin.

For the production of kaempferol from tyrosine, the expression of GmF3H and CuFLS was tested from a range of promoter combinations and operon configurations (Figure 3A). Due to the number of expression construct combinations, we expressed the genes from just the p15A plasmid backbone, which was shown to be more effective than expression from the pSC101 plasmid for the flavone synthases for apigenin production. Kaempferol production from tyrosine (Figure 3B.i) exceeded that of apigenin from tyrosine in three strains. Titers of kaempferol reached 528 ± 27 μM (151 ± 7.7 mg L^−1^), 515 ± 50 μM (147 ± 14 mg L^−1^) and 476 ± 76 μM (136 ± 22 mg L^−1^) for GmF3H and CuFLS expression from SBC015894, SBC015888 and SBC015892, respectively. In both SBC015888 and SCB015894, GmF3H and CuFLS were expressed from their own *lacUV5* promoter, with the only difference between the plasmids being the order in which the F3H and FLS expression cassettes appeared in the plasmid. While the use of the *trc* promoter was important for effective expression of PcFNS-I for apigenin production and use of the *lacUV5* promoter instead resulted in lower apigenin titers, expression of F3H or FLS from *trc* promoters often led to lower titers of kaempferol than comparable expression from *lacUV5*, e.g., with plasmid SBC015883 compared to plasmid SBC015892, and plasmid SBC015884 compared to plasmid SBC015894. Notably, the same chemical transformation catalyzed by F3H enzymes on naringenin to form dihydrokaempferol could be applied to eriodictyol to form dihydroquercetin, and the same chemical transformation catalyzed by FLS enzymes on dihydrokaempferol to form kaempferol could be applied to dihydroquercetin to form quercetin. Due to the structural similarities between naringenin and eriodictyol, as well as between dihydrokaempferol and dihydroquercetin, we also tested the same constructs for the expression of GmF3H and CuFLS as above for kaempferol production, but in strains with upregulated eriodictyol production rather than upregulated naringenin production. However, we did not detect any quercetin production in the resulting strains.

Titers of apigenin and kaempferol in Δ*tyrR* Δ*pheLA* knockout strains with upregulated tyrosine production from the SBC005753 plasmid, in cultures that were not supplemented with additional tyrosine, were also considerable (Figure 3B.ii). The highest apigenin titer reached with this host strain was 297 ± 31 μM (80.2 ± 8.4 mg L^−1^), achieved with the expression of LjFNS-II from plasmid SBC015677, while expression of SbFNS-II yielded a similar titer of 284 ± 45 μM (76.7 ± 12 mg L^−1^). The highest kaempferol titer in the same host strain was 212 ± 0.36 μM (60.7 ± 0.1 mg L^−1^), which was achieved by the expression of GmF3H and CuFLS in a single operon from the *lacUV5* promoter, on plasmid SBC015886. Moreover, the combined kaempferol and dihydrokaempferol titer reached by cultures of this strain was 259 ± 2 μM, which is 87% that of the highest apigenin titer in the same host strain.

Metabolic flux to chrysin and to luteolin was lower than expected and did not exceed titers previously reported in the literature (Table 1). Notably, chrysin was produced to a maximum titer of 6.8 ± 1.9 mg L^−1^ in rich media supplemented with a further 3 mM phenylalanine (Figure 3C.i), and luteolin was produced to a maximum titer of 5.0 ± 0.18 mg L^−1^ from 3 mM caffeic acid (Figure 3C.ii), with both of these titers being reached with the expression of GmFNS-II from plasmid SBC015675. While these titers are very low, the conversion of precursors pinocembrin and eriodictyol into chrysin and luteolin was high in some instances, and the rate of pinocembrin and eriodictyol production may thus be a contributing factor, if not the limiting factor. Notably, the production of pinocembrin by *E. coli* NEB5α SBC006524 (without further plasmids), reached only 50 ± 6.4 μM (12.8 ± 1.6 mg L^−1^) (Figure 3C.i, NEP), which is over 20-fold lower than the titer of naringenin reached by feeding *E. coli* NEB5α SBC006456 with 3 mM tyrosine. In contrast, Dunstan et al. (2020) were able to produce 198 mg L^−1^ pinocembrin with *E. coli* NEB5α SBC006524 grown in rich media supplemented with both phenylalanine (3 mM) and cerulenin. Similarly, they also produced 88 mg L^−1^ eriodictyol in cultures of *E. coli* MG1655 cells expressing Gm4CL, AtCHS and AtCHI, supplemented with caffeic acid (3 mM) and cerulenin. These results suggest that the addition of cerulenin could enhance the availability of (2*S*)-flavanone precursors, potentially leading to increased titers of chrysin and luteolin.

## 4 Discussion

### 4.1 Effectiveness of type II flavone synthases in *E. coli*

This study was successful in extending the naringenin pathway to produce high titers of apigenin and kaempferol, with apigenin titers being, to our knowledge, the highest reported in the literature for batch cultures of *E. coli*. The high titers achieved for apigenin production using type II flavone synthases were particularly surprising, as the good performance of this P450-dependent type of synthase was contrary to our initial expectations. This is noteworthy considering that the functional expression of plant cytochrome P450s and CPR in prokaryotes is typically challenging. Previous studies have demonstrated the complexity of post-translational modifications that are required for functional expression of cytochrome P450s, as well as issues related to protein insolubility due to solvent-exposed hydrophobic regions, since both plant cytochrome P450s and CPR are typically associated with membranes (Hausjell et al., 2018). Other studies have reported a lack of P450-reductase function (Nelson et al., 1993) and the need for supplementation of the growth media with 5-ALA to overcome limitations in heme biosynthesis (Gallagher et al., 1992; Sinha and Ferguson, 1998).

In our study, CPR from *A. thaliana* was effective overall, since four out of five FNS-II enzymes successfully produced apigenin, chrysin and luteolin. Only GhFNS-II did not produce notable titers of the three flavones, and this could be attributed to either poor protein expression levels, inactivity of this enzyme or AtCPR being a poor redox partner for GhFNS-II. Among the FNS-II enzymes, LjFNS-II achieved the highest apigenin titer, despite a significant amount of naringenin remaining unconverted. This partial conversion could be due to imbalanced expression levels of the enzymes in the naringenin pathway relative to LjFNS-II—a possibility, given the limited conversion of naringenin to apigenin by the benchmark PcFNS-II—or due to impaired cofactor recycling. In the latter scenario, alternative CPR enzymes could serve as better redox partners than AtCPR, and further studies may help to identify a more suitable CPR for LjFNS-II. Furthermore, the combined titers of apigenin and naringenin from strains expressing alternative FNS-II enzymes (plasmids SBC015673–SBC015676) were markedly lower compared to all other strains in the experiment, despite some of the enzymes exhibiting high turnover rates for naringenin. Conducting a time-course analysis of growth and production for these strains could provide valuable insights into the underlying cause.

Finally, we supplemented 5-ALA to ensure that there were no limitations in the availability of 5-ALA for heme cofactor production (Gallagher et al., 1992; Verderber et al., 1997; Sinha and Ferguson, 1998), but we did not perform experiments in the absence of 5-ALA to determine the functionality of FNS-II enzymes under such conditions. However, considering the cost constraints in an industrial bioprocess, supplementation of 5-ALA would not be feasible. As a result, alternative synthetic biology routes to upregulate heme production in *E. coli* (Verderber et al., 1997; Zhang et al., 2015) could be implemented to enable the catalytic activity of FNS-II enzymes in *E. coli* without the need for additional supplementation of 5-ALA to growth media.

### 4.2 Precursor supply limits the production of chrysin and luteolin

In our production assays, we observed low titers of chrysin and luteolin, accompanied by low titers of their precursors pinocembrin and eriodictyol in the control strains that lack FNS enzymes. Interestingly, we noticed a similar conversion yield pattern among both groups of FNS enzymes for all three flavones (compare Figure 3B.i, 3C.i and 3C.ii). Notably, the high titers of apigenin were achieved due to the abundant precursor supply in the naringenin chassis strain. This observation suggests that increasing the metabolic flux towards pinocembrin and eriodictyol could potentially lead to higher titers of chrysin and luteolin, respectively. One possible approach to boost the pinocembrin and eriodictyol titers is through cerulenin supplementation. However, it is important to note that this strategy would be costly for high-throughput screening assays and impractical for industrial-scale bioproduction.

A synthetic biology option to improve flux to pinocembrin would be to replace AtCHS with CHS from *Camellia sinensis* (CsCHS), as this enabled strains to produce 4 times more pinocembrin than the AtCHS equivalent when no cerulenin was added (Dunstan et al., 2020). Alternatively, Leonard et al. (2007) produced 429 mg L^−1^ pinocembrin in *E. coli* without cerulenin supplementation, by expressing 4CL from *P. crispum*, CHS from *Petunia hybrida*, and CHI from *Medicago sativa* in a strain engineered to increase malonyl-CoA availability from inexpensive precursors. Malonyl-CoA availability was increased by co-expression of an acetyl-CoA carboxylase (ACC) and an acyl-CoA synthetase, while co-feeding acetate with glucose. Moreover, when expressing just the ACC in the strain engineered to express Pc4CL, PhCHS and MsCHI, pinocembrin was produced to a titer of 230 ± 2 mg L^−1^, which is still considerably higher than the titers that we observed in our strains without cerulenin supplementation.

A synthetic biology approach to improve the flux to eriodictyol could involve the use of an alternative metabolic pathway to the one which we used in this study. For example, Zhu et al. (2014) produced eriodictyol in *E. coli* to 107 mg L^−1^ from tyrosine via naringenin by constructing and expressing a fusion protein (tF3′H-tCPR) of the cytochrome P450 enzyme flavanone 3′ hydroxylase (F3′H) from *Gerbera hybrida* and cytochrome P450 reductase from *Catharanthus roseus*. This fusion protein hydroxylates naringenin to form eriodictyol, and in the study by Zhu et al. (2014), naringenin was overproduced by co-expression of TAL from *Rhodotorula glutinis*, 4CL from *P. crispum*, CHS from *Petunia x hybrida*, CHI from *M. sativa*, and feeding tyrosine. Malonyl-CoA availability was also increased by upregulating ACC from *Corynebacterium glutamicum* ATCC 13032 and acetyl-CoA synthase from *E. coli* BL21, as well as disrupting the acetate competition pathway via deletion of *ackA*. Interestingly, this is another example of functional expression of a cytochrome P450 enzyme in *E. coli*, and as was done in this study, the authors supplemented the cultures with 5-ALA as a heme precursor to increase cytochrome P450 activity.

## 5 Conclusion

This study demonstrates the successful utilization of a biosynthesis pathway prototyping pipeline, involving retrobiosynthetic pathway design, automated enzyme selection, combinatorial pathway assembly and testing to extend the gatekeeper flavonoids pathways in *E. coli* to produce apigenin, chrysin, luteolin and kaempferol. The titers of apigenin are amongst the highest achieved in the literature for low-cell density batch cultures but did not require additional optimization to increase the availability of malonyl-CoA. Additionally, the kaempferol titers are, to our knowledge, the highest achieved in *E. coli*, though considerably lower than those achieved in *S. cerevisiae*, which is well known for its ability to express plant proteins better than many prokaryotic hosts can. While the titers of chrysin and luteolin are comparatively lower than apigenin, they are consistent with previous reports in the literature. These findings can be attributed to the constrained metabolic flux towards their respective precursors, pinocembrin and eriodictyol. Further improvements in metabolic flux towards precursor flavanones have the potential to considerably enhance flavone and flavonol production. This can be achieved by employing alternative host strains that offer increased availability of malonyl-CoA.

## Data Availability

The datasets presented in this study can be found in online repositories. The names of the repository/repositories and accession number(s) can be found in the article/Supplementary Material.
