# Supplementary material for: Expanding flavone and flavonol production capabilities in Escherichia coli
Source: Front Bioeng Biotechnol. 2023 Oct 18;11:1275651. doi: 10.3389/fbioe.2023.1275651 (PMC10619664; doi:10.3389/fbioe.2023.1275651)
Supplement: Supplementary file 1 [file DataSheet1.PDF]

Supplementary Material

1 Supplementary Figures and Tables

1.1 Supplementary Figures

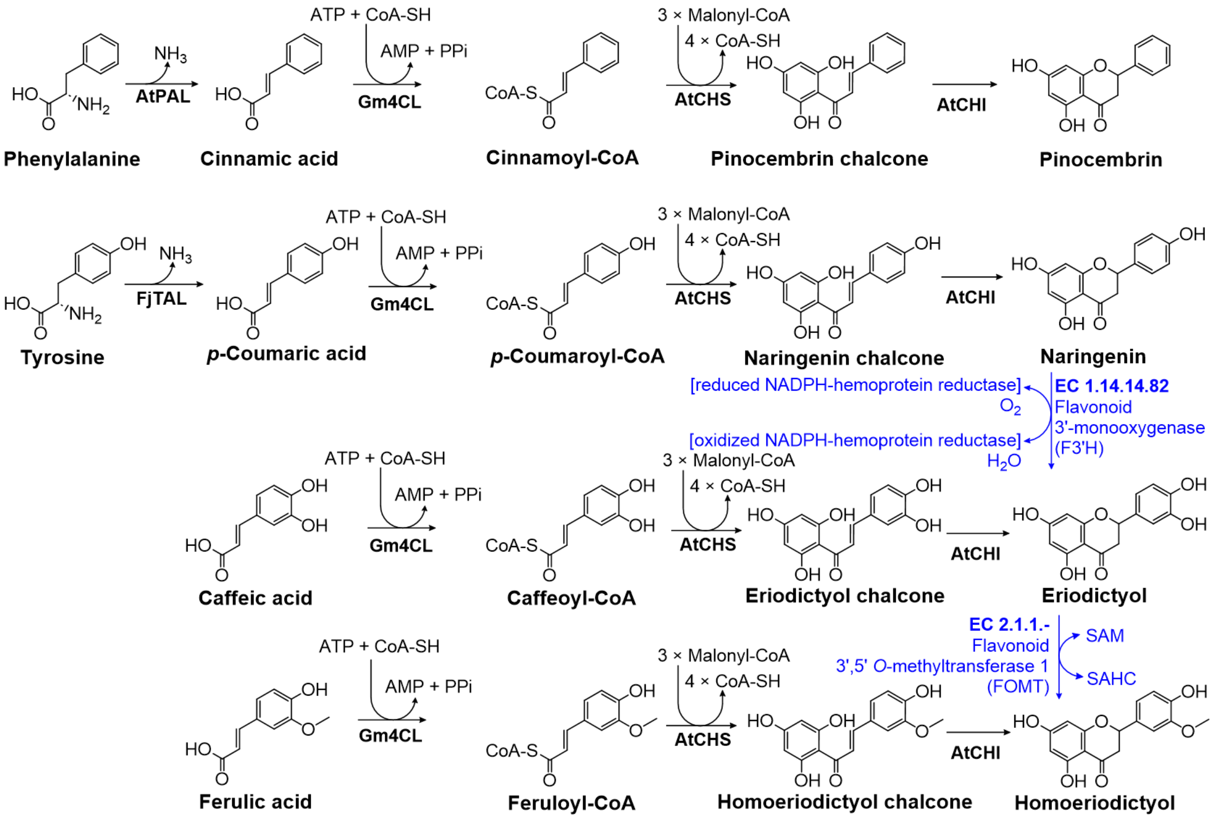

Supplementary Figure S1: Biosynthetic routes to gatekeeper flavonoids employed by Dunstan *et al.* (Dunstan et al., 2020). AtPAL: phenylalanine ammonia lyase from *Arabidopsis thaliana*; FjTAL: tyrosine ammonia lyase from *Flavobacterium johnsoniae*; Gm4CL: 4-coumarate-CoA ligase from *Glycine max*; AtCHS: chalcone synthase from *Arabidopsis thaliana*; AtCHI: chalcone isomerase from *Arabidopsis thaliana*. In blue: Alternative enzymatic routes to eriodictyol and homoeriodictyol more commonly cited as being the natural routes to these compounds.

1.2 Supplementary Tables

Supplementary Table S1: Plasmids used in this study that had previously been constructed by Dunstan *et al.* (Dunstan et al., 2020).

Plasmid SBC number	Antibiotic marker	Description
SBC005753 (pTyr+)	Km^R	Upregulation of tyrosine
SBC006456	Cm^R	Formation of naringenin from tyrosine
SBC006524	Km^R	Formation of pinocembrin from phenylalanine
SBC006845	Km^R	Formation of eriodictyol from caffeic acid

Supplementary Table S2: Sequences of the synthesized DNA parts. Gene coding sequences are shown in uppercase letters. EcoRI and BamHI restriction enzyme recognition sites are italicized.

Gene	Sequence
GmF3H	*gaattc*aaaagatctgagtcttgtaaggcaagggctcaattgagccccattttgtcgtatatacagtttcga<br>acccggaattgaatacagaggaggaaagacgtacaATGGCTCCAACCGCTAAGACCCTGACATATTTAGCCC<br>AGGAGAAAACATTAGAATCAAGTTTTGTACGAGATGAAGAGGAACGCCCGAAGGTGGCTTATAACGAATTTT<br>CAGATGAGATCCCGGTCATTTCCCTGGCCGGCATTGACGAAGTGGATGGCCGTCGGCGCGAAATTTGTGAGA<br>AAATCGTTGAGGCCTGCGAGAATTGGGGTATCTTCCAAGTAGTGGATCACGGCGTGGATCAACAACTGGTCG<br>CAGAAATGACGCGCTTAGCCAAAGAATTTTTCGCGCTTCCGCCCGATGAAAAATTACGATTCGATATGTCCG<br>GTGCAAAGAAAGGCGGCTTTATTGTGTCTTCACATCTGCAGGGCGAATCAGTTCAAGACTGGCGTGAAATCG<br>TTACGTACTTCTCGTATCCTAAACGTGAGCGTGATTACTCACGCTGGCCCGATACACCCGAAGGATGGCGTA<br>GCGTGACCGAGGAATATAGCGACAAAGTTATGGGGCTGGCCTGTAAATTAATGGAAGTACTGTCGGAGGCCA<br>TGGGCCTGGAGAAAGAAGGCCTGAGCAAAGCGTGTGTGGACATGGATCAGAAAGTAGTGGTCAATTATTACC<br>CCAAATGTCCTCAGCCGGATTTAACGCTAGGTCTGAAACGCCACACCGATCCGGGCACCATTACGTTGCTGC<br>TTCAGGACCAGGTTGGGGGATTGCAGGCGACACGCGATAACGGTAAAACTTGGATAACGGTCCAGCCGGTAG<br>AAGCGGCCTTTGTGGTTAACCTGGGCGACCACGCGCACTATCTGTCAAATGGTCGCTTTAAGAACGCAGATC<br>ATCAAGCTGTTGTTAACTCAAATCATAGCCGCCTTTCGATTGCCACCTTTCAGAATCCGGCGCCTAACGCGA<br>CCGTTTATCCGCTTAAAATCCGCGAGGGCGAAAAGCCGGTGATGGAAGAACCCATCACGTTCGCAGAAATGT<br>ATCGCCGTAAGATGAGTAAAGATATTGAAATTGCCCGGATGAAGAAATTAGCCAAAGAAAAGCATCTGCAGG<br>ATTTAGAGAATGAAAAACATCTGCAGGAACTGGATCAGAAAGCGAAGTTAGAAGCAAAACCGCTGAAAGAAA<br>TTCTTGCCTAAttgtagactc*ggatcc*
OsF3H	*gaattc*aaaagatctgagtcttgtagtttttaggcccgacatccccttgttaaactgaaaataaaacgttaa<br>cgtatgtaccctagaaactaacactgaaggaggttttcATGGCCCCTGTTGCGACTACCTTCCTGCCGACAG<br>CATCCAACGAGGCCACGCTCCGCCCGTCTTTTGTGCGCGATGAAGACGAGCGCCCTCGTGTGGCTTACAATC<br>AGTTTAGCGACGCGGTTCCGGTGATCTCACTGCAAGGAATTGATGAAGCAGCCCGGGCAGAGATCCGAGCCC<br>GTGTTGCTGGTGCTTGCGAGGAATGGGGCATCTTTCAGGTTGTCGATCACGGCGTTGATGCGGGCTTAGTAG<br>CAGATATGGCGCGCTTGGCACGCGATTTCTTTGCCCTGCCTCCGGAAGATAAACTGCGTTTTGATATGTCGG<br>GCGGCAAAAAGGGCGGTTTTATCGTGAGCTCACATCTGCAAGGCGAAGCCGTTAAAGACTGGCGCGAAATTG<br>TGACCTACTTCAGCTACCCGGTCAAAAGTCGCGATTATAGCCGTTGGCCTGACAAACCTGCGGGCTGGCGTG<br>CAGTAGTCGAGCAGTACTCTGAACGCCTGATGGGCTTAGCGTGTAAACTGCTAGGCGTCCTTTCAGAGGCAA<br>TGGGGCTTGATACTAACGCCCTGGCCGATGCATGTGTGGATATGGATCAGAAGGTAGTTGTCAATTTTTATC<br>CTAAGTGCCCGCAGCCCGATTTGACATTGGGGTTGAAACGTCACACCGATCCGGGCACCATTACCTTATTGT<br>TACAAGATTTAGTGGGCGGTCTGCAGGCGACGCGCGATGCTGGTAAAACGTGGATTACGGTACAACCGATTC<br>CAGGGAGCTTCGTTGTTAATCTGGGCGATCATGCCCATTATTTAAGCAACGGTCGTTTCAAAAATGCAGACC<br>ACCAGGCTGTAGTGAACTCGGACTGCTGCCGTTTAAGCATTGCAACCTTCCAGAATCCGGCCCCGGATGCTA<br>TGGTATATCCGCTGGCCGTTCGGGATGGAGAAGAACCGATTCTGGAAGAACCGATCACTTTTGCGGAGATGT<br>ACCGTAGAAAAATGGCCCGCGATTTGGAGCTGGCGAAACTGAAAAAGAAGGCAAAAGAACAGCGCCAGCTCC<br>AGCAGGCCGCACTCCCACCACCTCCGCCGACCCAGGTGGCCGCCGAGCTGGCTGCGCAGAAACCGAAAAGTC<br>TCGATGAAATCCTGGCGTGAttgtagactc*ggatcc*
PhF3H	*gaattc*aaaagatctgagtcttgtaccacctataccaatttcccaaatccaagcttacggtcaagataagaa<br>aatcaccttcctgatgaatgaacggagctaaaggaggtcccatctaATGATCCCGCGCGTGACCCCTTCAAC<br>CTTAACGGCCTTAGCGGAAGAGAAGACCCTTCAAACGAGCTTCATTCGCGACGAAGACGAACGCCCGAAAGT<br>GGCATACAATCAGTTTAGCAACGAGATCCCGATTATTTCATTGGAGGGCATTGATGATGAGACAGGGAAACG

AGCCGAAATCTGCGATAAAATAGTCAAGGCCTGCGAGGACTGGGGCGTTTTTCAGGTAGTGGATCACGGTGT
TGATGCAGAAGTGATTAGTCAGATGACAACGTTCGCGAAAGAATTTTTCGCACTGCCACCTGAAGAGAAGTT
ACGCTTCGACATGAGCGGCGGTAAAAAGGGTGGTTTCATCGTGAGTTCGCACCTACAGGGAGAAGTCGTGCA
GGATTGGCGTGAAATTGTAACCTATTTCTCCTATCCCACCCGCGCGCGCGATTACTCTCGTTGGCCGGATAA
ACCGGAAGGCTGGATTGCGGTGACGCAGAAATATAGCGAAAAGTTAATGGAGCTGGCCTGCAAACTGCTGGA
TGTGCTGTCGGAAGCCATGGGCCTGGAGAAAGAAGCCCTGACGAAAGCGTGCGTTGATATGGATCAGAAAGT
CGTTGTGAACTTTTATCCGAAATGCCCGGAGCCGGATCTGACACTGGGCCTGAAACGCCATACCGATCCGGG
CACCATTACACTGTTACTGCAGGATCAGGTGGGCGGATTGCAGGCGACCAAGGATAATGGGAAAACCTGGAT
AACGGTGCAACCGGTTGAGGGAGCCTTTGTTGTGAATCTTGGTGACCACGGCCATTTCTTGAGCAACGGTCG
CTTTAAAAACGCCGATCATCAAGCGGTGGTGAACTCAAATAGTAGCCGTCTGAGCATTGCAACATTTCAGAA
CCCTGCGCCAGAAGCTATTGTCTATCCTCTAAAAATCAGGGAAGGCGAAAAGTCCATCATGGATGAACCCAT
TACATTTGCGGAAATGTATCGTCGCAAAATGTCTAAAGACCTCGAACTGGCTCGCTTGAAGAAACAAGCAAA
AGAACAGCAACTGCAAGCAGAAGTGGCTGCCGAAAAAGCCAAGCTCGAAAGTAAACCGATTGAAGAAATACT
GGCATGAttgtagactc*ggatcc*

CsF3H

*gaattc*aaaagatctgagtcttgtaacaataaactgtgagttctacttgaaaatcgccgctgagatagtact
ttagattattgccagggttacttgacaccaaagaagaaggagggaatgATGGCTCCTAGCACTCTGACAGCG
CTCGCCGGCGAAAAGACGCTAAACCCGAGTTTCGTTCGATTCCAGGACGAACGCCCGAAAGTTGCGTATAAT
GAATTTAGCAACGAAATTCCGGTAATTTCGTTGGCGGGGATTGATGATGTCGGTGGTAAACGTGCAGAAATC
TGCAAAAAGATTGTAGAAGCATGTGAGGATTGGGGCATTTTCCAGGTAGTCGATCACGGGGTGGATGCAAAA
CTGATCTCGGACATGACCCGGTTAGCCACGGAGTTCTTCGCCCTACCTCCGGAAGAAAAACTGAAATTCGAT
ATGTCAGGTGGCAAGAAGGGCGGCTTTATCGTTAGCAGCCACCTGCAAGGTGAGGTGGTGAAAGACTGGCGC
GAAATCGTAACCTATTTTAGTTTTCCGAAACAGAGCCGTGATTATTCGCGTTGGCCGGATAAGCCAGAGGGC
TGGATGGAGGTGACGAAAGAATACTCAGACAAACTTATGGGGGTCGCGTGTAAATTGCTGGAAGTTTTGAGC
GAAGCAATGGGCTTGGAGAAAGAAGCCTTAACCAAGGCGTGTGTGGATATGGATCAGAAGATTGTAGTGAAC
TACTACCCTAAGTGTCCGCAGCCTGACCTGACCCTGGGTCTTAAACGCCATACTGACCCGGGTACCATTACC
CTGTTACTGCAGGATCAGGTGGGTGGCCTCCAGGCGACTAAAGATAATGGCAAAACATGGATTACCGTGCAA
CCCATTGAAGGTGCGTTTGTTGTGAACCTCGGCGATCACGGACACTATCTGTCGAATGGTCGGTTTAAAAAC
GCGGACCACCAGGCCGTTGTAAACTCTAATTCAAGTCGTCTGAGTATTGCCACCTTTCAGAACCCTGCTCCG
GAAGCGACCGTTTACCCTCTGAAGATTCGCGAAGGAGAAAAGCCAGTCCTGGAAGAGCCTATTCCGTTTTCG
GAAATGTACCGTCGCAAAATGAGTAAGGATCTTGAGCTGGCACGCCTGAAGAAATTAGCCAACGAGAAGAAA
CAGTATTCCGAAAAAGCCAAACTGGACGCTAAACCTATTGAAGAAATACTAGCGTAAttgtagactc*ggatc*
c

CuFLS

*gaattc*aaaagatctgagtcttgtagtgacatcttagaagattagacacagaagttggaggacaaacgcgtg
accacccgctggggaagtcatgtggtaactaatgatgaggaggtatataATGGAAGTAGAACGTGTCCAGGC
CATCGCGAGCCTGTCTCATAGCAACGGAACCATTCCGGCGGAATTTATTCGTCCGGAAAAGGAACAGCCGGC
GTCCACCACGTATCATGGCCCGGCGCCGGAGATTCCAACCATCGATCTTGACGACCCGGTCCAGGACCGCCT
GGTCAGGAGCATTGCGGAAGCGTCACGTGAGTGGGGCATCTTTCAAGTGACTAATCATGGGATTCCTAGCGA
TTTAATCTGCAAACTGCAGGCGGTAGGCAAAGAGTTCTTTGAACTCCCGCAGGAAGAAAAGGAGGTCTACTC
GCGCCCCGCGGACGCTAAGGATGTACAGGGCTATGGCACCAAACTGCAAAAAGAAGTTGAAGGCAAAAAGTC
ATGGGTTGACCACCTCTTCCATCGCGTTTGGCCGCCATCGAGTATTAACTACCGTTTCTGGCCGAAAAACCC
ACCGAGTTATCGCGCAGTGAATGAAGAATACGCTAAATACATGCGCGAGGTCGTTGATAAACTGTTTACATA
TCTGTCACTGGGCCTTGGCGTCGAAGGCGGCGTCTTAAAAGAAGCAGCGGGTGGTGATGATATCGAATACAT
GTTGAAAATTAATTATTATCCGCCATGCCCGCGCCCTGACCTGGCGCTGGGTGTGGTGGCGCATACCGACTT
ATCAGCCCTAACCGTGCTCGTTCCAAACGAAGTGCCGGGCTTGCAGGTATTTAAGGATGATCGGTGGATTGA
TGCGAAATATATTCCGAATGCCTTGGTTATTCATATTGGCGATCAAATTGAAATTTTAAGCAACGGCAAATA
CAAAGCGGTCCTGCATCGCACAACCGTGAACAAAGATAAAACACGCATGTCGTGGCCGGTATTTCTGGAACC
TCCTGCGGATACCGTTGTTGGCCCACTGCCTCAACTGGTCGACGATGAAAATCCGCCTAAATACAAAGCCAA
AAAGTTTAAAGACTACAGCTACTGTAAACTGAATAAACTACCGCAGTGAttgtagactc*ggatcc*

CsFLS

*gaattc*aaaagatctgagtcttgtaagagcttgagtgttacatgtgttcaacactgacacttcttccccaag
aaaagaaaacctgccgtgccctcgcggaggaactaaggaggattgATGGAAGTAGAACGCGTTCAGGCCTTA
TCACACGTAACCCTGCATGAACTGCCAGCGAAATTCATTCGCCCGGTGCATGAACAGCCGGAAAATTCTAAA
GCGATTGAGGGTGTGACCGTGCCGGTTATTTCCTTAAGCCAACCACATGATGTGGTGGTGGATGCCCTGAGC
AAAGCCTGCTCGGAATGGGGTTTCTTCCTGATCACCGATCATGGAGTTGAGCCGTCGCTGATCGGCCGTCTG
AAGGAAGTTGGCGAAGAATTTTTCAACTTGCCACAAAAAGAAAAAGAGAGCTACGCAAACGACCCGTCATCG
GGTTCATTCGAGGGATACGGCACCAAGATGACCAAAAACTTCGATGAAAAAGTCGAATGGATCGATTACTAC
TTTCATGTAATGCATCCTCCTAAAAAGCTTAATCTGGACATGTGGCCCAAAAATCCGTCCTCGTACCGTGGA
GTGACCGAAGAATACAACGTTGAAATCATGCGGACCACAAACAAACTGTTCGAACTGCTCAGCGAGGGTCTG
GGCCTGGATGGTAAAGTGTTATCTTCAAGCCTGGGCGGCGATGAGATCGAGTTCGAAATGAAAATTAACATG
TATCCGCCGTGCCCGCAGCCTCAGTTAGCACTGGGGGTTGAACCGCATACTGACATGAGCGCCTTGACGTTA
TTAGTTCCGAACGACGTTCCTGGTTTACAGGTGTGGAAAGATGGTAACTGGGTGGCAGTGAATTATCTCCCG
AACGCCCTGTTTGTGCATGTTGGCGATCAGTTAGAGGTGCTGAGCAATGGCAAATACAAGTCGGTTCTGCAC
CGCAGCTTGGTAAACAAAGAACGTACCCGCATGAGCTGGGCCGTGTTTGTGGTTCCGCCGCATGAAGCGGTT
ATTGGCCCTCTTCCGGAATTGATTGATGAGAAAAATCCGGCAAAATATTCAACAAAAACGTATGCAGAATAT
CGTCACCGTAAATTTAATAAGATCCCACAGTAAttgtagactc*ggatcc*

GbFLS

*gaattc*aaaagatctgagtcttgtagaggactgattgcgtcttactaatgcctatcctaatccgagtggtta
gacatcagaatatgagaaatcaagatcccggagggtaggtaataaATGGCCCCAACGCGTGTTCAATATGTC

GCCGAATCCCGCCCGCAGACGATCCCTCTGGAATTTGTGCGTCCTGTTGAAGAACGTCCTATTAACACGACC
TTCAACGACGATATTGGACTGGGCCGCCAAATTCCCGTAATCGATATGTGCAGCCTGGAAGCACCGGAGCTT
CGCGAGAAAACGTTTAAAGAGATCGCACGCGCATCCAAAGAATGGGGAATTTTTCAGGTGATTAATCACGCC
ATTTCACCGTCGCTGTTCGAAAGTCTTGAAACTGTTGGAAAACAATTCTTTCAGCTCCCCCAAGAGGAGAAG
GAAGCTTACGCCTGCACAGGTGAAGACGGCAGCTTTACAGGGTACGGCACTAAGTTGGCGTGTACCACTGAC
GGACGTCAAGGCTGGTCTGACTTCTTCTTTCACATGTTATGGCCGCCGTCCCTGCGAGATTTTTCGAAATGG
CCGCAAAAACCATCGAGTTATATTGAGGTAACAGAAGAATACTCCAACCGCATTCTGGGCGTTTTGAATAAG
CTGCTGAGCGCACTGTCAATCAGCTTGGAACTGCAGGAAAGTGCGCTGAAAGATGCGTTAGGGGGAGAAAAC
CTCGAAATGGAGTTAAAAATTAACTATTACCCGACCTGTCCGCAGCCCGAGGTTGCGTTTGGCGTGGTGCCG
CACACCGATATGTCGGCCCTGACCATCCTGAAACCGAACGATGTGCCAGGCCTTCAGGTCTGGAAAGATGAT
AAATGGATTACGGCTCACTATGTGCCTAACGCCTTGATCATTCACATCGGAGATCAAATTCAGATTTTATCA
AATGGTAAATTTAAAAGCGTTCTGCATCGTAGTTTAGTAAACAAAGAAAAAGTGCGTATGAGCTGGCCGGTT
TTCTGTAGCCCGCCGCTAGACACCGTGATTGGTCCGCTGAAAGAGTTGATTGATGATAGCAATCCGCCGTTG
TACAATGCGAAAACCTATCGCGAGTACAAGCACCGAAAAATTAACAAATTAGGTCAATAAttgtagactc*gg*
atcc

ZmFLS *gaattc*aaaagatctgagtcttgtaaggcacgttaacatgagtatccactcgaacttacgcgcgcattccat
cgtacaagtggggacgtacgagggagaggaggaagcatacgATGGGCGGTGAAACACATTTATCTGTTCAGG
AACTAGCGGCGTCGCTGGGCGCGCTACCGCCTGAGTTTGTACGCAGCGAACAGGACCAGCCGGGTGCGACCA
CCTACCGCGGCGCCGCGGTACCTGATGCGCCTGTAATTGACATCAGCGAACCTGGATTCGGCGCACGCATGG
CGGCGGCTGCTCGCGAATGGGGCCTGTTTCAGGTGGTAAACCACGGGGTGCCTAGTGCGGCAGTGGCGGAAC
TCCAGCGTGTGGGCCGCGCGTTCTTTGCCCTGCCGACGGAAGAAAAAGAACGCTATGCCATGGACCCCGCGT
CGGGGAAGATAGAGGGCTATGGCACAAAACTGCAACGCGACTTAGAAGGAAAGAAAACCTGGAATGACTTTT
TCTTCCACGTGGTTGCACCGCCGGAAAAAGTTGATCACGCAGTCTGGCCACGCAGTCTGGCCGGTTACCGCG
AAGCGAATGAAGAGTATTGTCGCCATATGCAGCGCCTGACCAGGGAGTTGTTCGAACATTTAAGCTTAGGCC
TTGGCCTTCATGGCGGCGCCATGGCCGAAGCTTTTGGTGGCGACGGCCTGGTGTTTTTACAGAAAATTAATT
TTTATCCGCCGTGCCCACAGCCTGAACTTACCCTGGGCGTTGCGCCCCATACGGACATGAGCACATTAACCG
TGCTTGTACCCAACGAGGTGCAGGGGCTGCAGGTTTTCAAAGATGGCCAGTGGTACGAAGCTAAGTATGTAC
CGGACGCCCTGATCGTGCATATCGGCGACCAGATTGAAATTTTCAGTAACGGCGCATACAAAGCCGTGCTGC
ATAGGACCACGGTGAACAAAGAGAAAACCCGCATGTCGTGGCCTATGTTCGTAGAACCACCGGGCGAACTGG
TTGTCGGGCCTCATCCAAAGCTTGTGACCGAAGAATCGCCGGCCAAATATAAAGCGAAAAAGTACAAAGATT
ACCAGCATTGCAAAATTAATAAGCTGCCTATGTAAttgtagactc*ggatcc*

PcFNS-I *gaattc*aaaagatctgagtcttgtagtagaccgctccgtgtgctaggaccaaagtagtgtgatgacgtcaag
ggcattgtacatctctgtcactaacaaggaggactaATGGCGCCTACCACGATCACTGCCCTCGCGAAAGAG
AAAACACTGAATCTGGATTTCGTTCGTGATGAAGATGAACGCCCGAAAGTCGCCTATAACCAGTTCAGTAAT
GAGATTCCAATTATCTCACTTGCGGGCTTGGATGACGACAGCGATGGTAGACGGCCGGAAATTTGCAGAAAG
ATAGTGAAGGCGTGTGAAGACTGGGGTATTTTTCAAGTGGTGGACCATGGTATTGATTCAGGATTAATTAGC
GAAATGACCCGCCTTTCCCGTGAGTTCTTTGCGCTTCCGGCAGAGGAGAAACTAGAGTACGATACGACGGGT
GGTAAGCGAGGCGGGTTTACTATTAGTACTGTGTTGCAAGGTGATGACGCAATGGATTGGCGCGAATTTGTG
ACGTACTTTAGCTACCCGATCAACGCTCGTGATTACTCGCGGTGGCCGAAAAAGCCTGAAGGCTGGCGCAGC
ACGACGGAAGTTTATAGCGAAAAACTGATGGTTTTAGGCGCTAAACTCCTTGAAGTCCTGAGCGAAGCGATG
GGCCTAGAGAAAGGCGATCTGACCAAGGCGTGCGTTGACATGGAACAGAAGGTTTTGATCAATTATTACCCT
ACGTGTCCGCAACCGGATCTTACCCTGGGCGTGCGTCGTCATACCGATCCGGGTACCATCACCATACTGCTC
CAGGACATGGTGGGAGGCTTGCAGGCGACGCGTGATGGCGGGAAAACCTGGATCACGGTGCAGCCGGTAGAA
GGCGCCTTCGTCGTGAATCTAGGCGATCATGGACACTATCTGTCAAACGGCCGCTTTCGCAATGCTGACCAT
CAGGCAGTCGTCAATTCGACGAGCTCTCGTTTGTCCATTGCCACCTTTCAGAACCCGGCGCAGAACGCCATT
GTTTATCCGCTGAAAATTCGAGAAGGCGAAAAAGCCATCCTGGATGAAGCGATAACATACGCCGAGATGTAT
AAGAAGTGCATGACAAAACACATCGAAGTAGCGACCCGTAAGAAACTGGCCAAGGAAAAACGCTTACAGGAT
GAAAAAGCTAAGTTAGAGATGAAAAGCAAAAGCGCGGACGAGAACCTGGCGTAAttgtagactc*ggatcc*

PaFNS-I *gaattc*aaaagatctgagtcttgtaaccgacgcgctgtgccagtcgagttaagtctcaccatactcacaacg
actagttaacgaaatagaggaggtatgagaATGGCCCCGCCAGCTGTAGCCGAGTTCCCCAATGACACGAAA
CCGGTTGTGGCGGCAAGCGTTATGAAACTGGCGAACGAACTCACCGTGTTACCGGAAAAATTTGTTAAAGCT
GTGGGCGAACGTCCGGTGACTGCGCACAACGACTACTGCAAAGAAATTCCAGTGATCAGCTTGAAGGGCATC
GATAATGAATCTGAACGGGCGCGTATCGTGGCGGAGGTAGGCCGCGCCTGTGCAGATTGGGGCATCTTCCAG
ATAGTGGACCACGGTATGTCCGCCGTCTTGATGAAACGCACTATGGAGACAGTGCTCGAATTTTTCAAACTG
CCAGTTGAGGAAAAGTTGAAATGTGCAGCAAAACCCGGGGACTTTCCGGTTGGCTATGCCTCAGGTTCTCAT
CGCGCATCGGATGATGTTCTTGACTGGCGTGAATTTATGGCGCATCGCAGCTTGCCTAAGGCCATCCGTGAA
AATGATATCTCGACCTGGCCGGACAAGCCGGAGCATTATCGAAAAACTCTGGTGGAATATTGCGACGCGTGC
GATGGGTTGGTTACTGCGCTGTTGGGCTTAATTAGCGAAAGTTTAGGCCTTCCGACGAATTACATCAAAAAC
TTTGTGGGTGGCGACGACGCGGACCAGAAAATGATTCTCAACTACTACCCGCAATGCCCTATGCCCGATCTG
ACCTTGGGCTTACGATCACATACCGACTACGGTACGATTACGGTGTTACAGCAGGATCAGGTCGGCGGACTT
CAAGTGTACAATGCAGACCGTCGCAAGTGGGTGACCGTCGAGCCTATTCCGGGTGCTCTGGTCGTGAATCTG
GCGGATCAGTTGCAAATTCTCAGCAATAATAAGTACTGTTCCGTGATGCACCAAGCCATCGTGAATAGCAAT
CATACTCGCCTGTCGATCGCCACGTTGAGTAACCCGAACGCCATGAGCCAAATGGGACCTGCCCCCGAACTG
TTATCGGCGGAAAACCCGGCGAAATACCGCACCTACAAATTCAAGGAATACCTGCCAATATGCATTGCGAAA
AAGGCGACAAATCATTGTGATGCCGTGGCGCTGTAAttgtagactc*ggatcc*

AtCPR2

*gaattc*aaaagatctgagtcttgtagcctgatgtccgtggagcaacctggtaggagactggtgatgtgataa
atcttgtacaaaccagtggaattaaggaggttctcATGCGTCGTAGCGGCTCCGGCAACTCAAAGAGAGTGG
AGCCCCTGAAACCATTAGTTATTAAACCACGTGAAGAAGAGATAGATGATGGGCGCAAAAAGGTGACCATTT
TCTTTGGTACGCAAACCGGAACTGCCGAAGGTTTTGCCAAAGCGCTCGGCGAAGAGGCTAAGGCCCGTTATG
AGAAAACCCGCTTTAAAATCGTTGACCTTGATGACTATGCAGCGGACGATGATGAGTACGAAGAAAAATTGA
AGAAAGAGGATGTGGCGTTTTTCTTCCTGGCCACGTATGGTGACGGCGAACCGACGGACAACGCGGCGCGCT
TCTATAAATGGTTTACCGAAGGAAATGATCGGGGCGAATGGCTGAAAAATCTTAAGTATGGCGTATTTGGTT
TGGGCAATCGTCAGTATGAACACTTTAACAAAGTGGCCAAAGTGGTGGATGATATTCTCGTTGAACAAGGTG
CACAGCGCCTGGTGCAGGTAGGGTTAGGCGACGACGATCAGTGTATCGAGGACGACTTTACGGCATGGCGTG
AAGCCCTGTGGCCGGAACTTGACACCATTCTACGTGAAGAGGGTGACACCGCCGTAGCTACGCCGTACACGG
CGGCCGTTTTAGAATACCGTGTCTCGATCCACGATTCCGAGGATGCAAAATTCAATGACATTAATATGGCGA
ACGGCAATGGATACACAGTTTTCGATGCTCAACACCCCTATAAAGCAAACGTGGCAGTTAAACGTGAGCTGC
ACACCCCGGAATCAGACCGTTCATGCATCCATTTAGAATTTGATATTGCAGGCTCTGGCCTGACGTATGAGA
CTGGCGACCACGTGGGGGTCTTATGTGACAATCTGTCCGAAACGGTCGACGAAGCACTACGCCTTCTTGACA
TGTCTCCCGATACCTACTTTAGCTTGCACGCGGAAAAGGAAGACGGTACGCCGATTTCGAGCAGCTTGCCAC
CCCCATTTCCTCCCTGTAATCTGCGTACCGCCCTGACGCGCTATGCGTGTCTGCTGTCAAGTCCGAAGAAGA
GCGCCCTCGTTGCTCTCGCCGCGCATGCGAGCGATCCTACCGAGGCGGAACGTCTAAAACACCTGGCGTCCC
CGGCGGGCAAAGACGAATATAGCAAATGGGTGGTGGAATCACAGCGTAGTCTGCTTGAAGTGATGGCCGAGT
TCCCATCCGCGAAACCTCCGCTGGGGGTGTTTTTCGCAGGCGTTGCGCCGCGCTTACAGCCTCGTTTCTATT
CAATTTCCAGCAGTCCCAAAATCGCGGAAACACGCATTCATGTTACCTGTGCCCTGGTGTACGAGAAAATGC
CTACGGGTCGTATTCACAAGGGTGTTTGTAGTACGTGGATGAAAAATGCAGTTCCGTATGAAAAATCGGAAA
ACTGTTCCTCAGCCCCGATATTTGTGCGTCAGTCTAATTTCAAACTACCGTCGGATAGTAAAGTGCCGATTA
TTATGATTGGACCAGGCACGGGCTTAGCGCCGTTCCGCGGGTTTCTCCAGGAAAGGCTTGCACTGGTAGAAT
CTGGTGTGGAACTTGGTCCGAGCGTGCTGTTCTTTGGATGCCGCAATCGTAGAATGGACTTTATTTACGAAG
AAGAATTACAGCGTTTTGTCGAAAGCGGCGCACTGGCGGAGCTGTCTGTCGCATTTAGCCGTGAAGGCCCCA
CGAAAGAGTACGTGCAGCATAAAATGATGGATAAAGCGTCTGATATCTGGAATATGATTAGCCAAGGCGCCT
ATCTGTATGTGTGCGGTGATGCGAAAGGCATGGCACGCGACGTACATCGTAGTTTGCACACGATCGCACAGG
AGCAGGGCAGCATGGATAGCACCAAAGCGGAGGGTTTCGTGAAAAATCTACAGACCTCAGGCCGCTACTTAC
GCGATGTGTGGTAAttgtagactc*ggatcc*

SbFNS-II

*gaattc*aaaagatctgagtcttgtagcacttaatggccatcctggggcttgcatttaccatattattgattc
atattcatcatagtccatatcaaggaggaaaatcagATGTCACCCCCAGGCCCGTTCCCTCTGCCGATTATC
GGGCACTTGCATCTGCTGGGCCCGAAGCTGCATCAGTCGTTCCATGGCCTTAGCCAGCGCCACGGCCCGCTT
ATGCAGATTCGCTTAGGGAGCATTAATTGCGTCGTAGCTTCGACACCAGAACTGGCGAAGGAGTTCCTAAAA
ACGAATGAACTGGTGTTCTCTAGCCGTAAACATAGTACTGCCATCGACATAGTGACCTATAATTCTAGCTTT
GCTTTTAGCCCATATGGACCATATTGGAAGTACATCAAGAAACTCTGTACATACGAGTTGCTGGGTGCCCGT
AACCTGCATCATTTTCAACCGATTAGGACGTTTGAGGTGCACACGTTCCTCAGACTGCTTATGGAGAAGAGC
GAGAGCGGCGAATCGTTTAATGTTACAGAAGAACTCATCAAACTGACCTCAAACGTTATGAGCAATATGATG
CTCGGTACGCGTTGCTCAGCGACCGATGGAGAAGCGGAAGCAGCACGCACAGTAATTCGCGAAGTTACTGAA
ATTTTTGGTGAGTTTGATGCGGCCGACATTATTTGGTTTTGTAAAAACTTCGACCTGCAGGGGATTCGTAAA
CGAAGCGAAGATATTCAGCGCCGCTACGATGCACTGCTGGAAAAGATTATCACCGATCGGGAGAAATTGCGC
CGTTCGCACCGCGGAGGTGAAGCAAAGGACTTTCTGGATATTTTCCTTGATATCATGGACAGTGGTAATTCC
GAAGTGAAATTCTCCCGCGAGCACCTGAAAGCGCTGATCCTGGATTTCTTCACGGCAGGCACCGACACTACG
GCAATTAGCACCGAATGGGCCATTGCGGAACTGATGAATAATCCCAAAGTATTAAAGAAAGCTCAGGAAGAA
ATTCAAAAAGTCGTGGGGAGCTGTCGTCTGATGGACGAATCTGATGCGCCGAACCTGCCGTACCTGGAAGCA
ATTATAAAAGAAACGTTTCGCTTGCATCCTCCTATCCCGATGCTGGCGCGTAAAAGCGTAAGTGATTGCGTG
ATCGACGGCTACAACATCCCGGCGTCGACCCTGTTATTCGTAAATATTTGGAGCATTGGACGCAACCCGGAA
TGCTGGGACAGCCCGTTTAGCTTCCGTCCGGAACGCTTCTTCGAGAAAGACAATGCCAGCATTGATATTAAA
GGACAACACTTTCAGTTGTTACCGTTCGGCACGGGCCGTCGCGGGTGCCCGGGCATGTTACTGGCGATTCAG
GAACTGCTGCTGATTATCGGAACCATGATTCAGTGTTTCGACTGGGAATTACCGGAGGGTTCTGGTCCGGTG
GATATGACCGAGCGTGCCGGCTTGACCGCCCCGCGTGCCGAAGACTTAATTTGTCGTGTGAGCTGTCGCGTT
GACCCAAAAATTGTGTTCTAAttgtagactc*ggatcc*

ObFNS-II

*gaattc*aaaagatctgagtcttgtaccgttaaggcaccatcgtactagaattgactaatcatctggatgact
tgatcactaaaagtaagttaatgagaatcgaagaggaggtcgaaATGGGTTATCGTCGCCGTACTTCATCGC
CGCCAGGCCCCTTTCCGCTGCCGATTATTGGTAATCTGCATTTGTTGGGTCCGCGCTTACATCACAGCTTTC
ATGATCTTACCAAAAAGTATGGTCCGCTGGTCCAGTTACGCCTGGGCTCTGCTTCATGCGTAGTGGCTGCTA
CACCGGAACTGGCGAAAGAGTTCCTGAAAACTAATGAACTGGTATTTTCAGCACGTAAACATAGTACTGCGA
TTGACATCGTTACGTACGAATCCTCATTTGCATTTGCTCCGTACGGGCCATATTGGAAATTTGTCAAAAAGC
TGTGCACATACGAATTGCTGGGTGCCCGTAACCTGAATCACTTTCTTCCGATTCGCAAACTTGAGGTTAAAA
CGTTCCTCGAAATTCTGATGCAGAAAGGGAAAATGGGTGAAAGCTTTAACGTTACAGAGGAGTTGATGAAAT
TAACCAGCAATGTAATCAGCCATATGATGCTGTCTATTCGATACAGCGGCACCGACGGCGAAGCTGAAGCCG
CACGCACGGTGATCCGTGAGGTAACCCAAATTTTCGGCGAATTTGACGTAGCAAACATTATTTGGTTTTGTA
AGAACTTCGACTTCCAGGGAATTAGAAAACGCAGCGAAGACATCCGACGTCGTTATGACGCGCTGCTGGAAA
AGATTATTACAGACCGGGAAAAGCTTCGACGTAGCCACGGTGGTGGCGGCGAAGCCAAAGACTTCCTGGATG
TTTTTCTGGACGTAATGGAATCGGGTAAAGCTGAAGTGAAATTCACGCGTGAACACCTGAAAGCGTTGATCC
TGGATTTCTTCACGGCGGGCACTGACACGACCGCGATTATCTCAGAATGGACCATCGCGGAACTGATTAATA
ACCCGACTGTTCTGAAAAAGGCCCAGACTGAAATTGACACTGTAGTAGGAGTTGACCGCCTCCTGCAAGAAT

CTGATGCCCCTAACTTGCCGTACCTGAATGCCATCATTAAAGAAACCTTCCGTCTGCATCCGCCTATACCGA
TGCTGAGCCGTAAAAGCACCAGTGACTGTGTGATTGGCGGCTACACCATCCCAGCGGATACGCTTTTATTTG
TAAATATTTGGTCAATGGGTCGAAATCCGAATATCTGGGAAAACCCTACCGAATTTCAGCCAGAGCGTTTCT
TAGAAAAGGAAAACGCCGCGATCGATATTAAAGGTCAGGATTTCGAGCTGCTGCCTTTTGGCACCGGCCGGC
GTGGGTGTCCGGGCATGCTGTTAGCCATACAGGAAGTTACGTCTGTTATTGGAACCATGATTCAATGCTTCG
ACTGGAAACTCCCGGCGGGGGACGGTTCGGATCGAGTGGATATGACCGAACGCCCGGGCCTCACCGCGCCGC
GCGCCGAGGACCTGGTTTGCTGCGTGGTGCCGCGTGTTGACGCCCTGGATGTAAGCTCGCTGTAAttgtaga
ctc*ggatcc*

GmFNS-II

*gaattc*aaaagatctgagtcttgtagcgataatgtgtctatgagaaaatgcgtgcccgcaattagtagttta
agaatgtcgatgataattggtccgagtaagttataggaggtttgATGAGTTTACTGAAATTGCTTTTCGTTC
GAGAGAACAAACCGAAAGCCCATCTTAAAAACCCGCCGAGCCCACCGGCAATCCCGATTATTGGTCACCTGC
ACTTACTGAAGCCGTTAATTCATCACAGTTTTCGCGACCTCAGCTTGCGGTATGGCCCACTTCTGAGCCTGC
GCATTGGCAGTGTCAAATTCATTGTCGCGTCAACACCTAGCCTGGCGCAGGAGTTTCTGAAGACCAATGAAC
TGACTTACAGCTCACGCAAAATGAATATGGCAATTAACATGGTAACCTACCATAATGCGACCTTTGCCTTTG
CCCCGTATGATACCTACTGGAAATTCATGAAAAAGTTATCTACCACCGAATTGTTAGGTAACAAAACGCTCG
GACATTTCTTGCCGATTCGTACCCGTGAAGTTCACGATATCATCCAGTTCTTGTTTCACAAAAGCAAAGCCC
AGGAATCCGTCAACCTGACCGAAGCCCTTTTGAGCCTGTCGAACAATGTGATTAGCCAGATGATGCTTTCGA
TTAAAAGTTCAGGGACCGATTCGCAGGCAGAACAAGCCCGTACATTAGTGCGCGAGGTGACCCAGATTTTTG
GAGAGTTTAATGTTTCAGACTTCCTTGGCTTCTGCAAAAATCTCGATCTGCAGGGCTTTCGCAAACGTGCGC
TGGATATTCATAAGAGGTACGATGCTTTACTAGAGAAAATCATTAGTGACCGCGAGGAGCTTCGCCGCAAAT
CGAAAGTTGATGGTTGTGAAGATGGAGACGACGAAAAGGTGAAAGACTTCCTCGATATCCTGCTGGATGTCG
CCGAGCAGAAGGAATGCGAAGTGCAGCTTACCCGCAACCACGTGAAATCTTTAATTCTCGATTATTTCACTG
CTGCCACAGACACAACCGCCATTTCGGTGGAATGGACGATCGCTGAACTCTTCAACAATCCGAAAGTGTTGA
AAAAGGCCCAGGAGGAAGTTGATCGAGTGACCGGCAACACGCAGCTTGTGTGTGAAGCCGACATCCCTAACC
TTCCGTATATTCATGCAATCATCAAAGAAACTATGCGTTTGCACCCGCCGATTCCGATGATCATGCGCAAAG
GCATCGAAGATTGCGTAGTCAACGGCAACATGATTCCAAAAGGCTCTATCGTATGTGTGAACATCTGGGCCA
TGGGTCGTGATCCGAACATCTGGAAGAATCCGTTAGAATTTAAACCAGAACGCTTCTTGGAGGGTGAGGGGA
GTGCAATTGATACCAAAGGTCATCACTTTGAACTGTTGCCGTTTGGTAGCGGCCGCCGTGGCTGCCCTGGCA
TGCCCCTCGCAATGCGCGAACTGCCTACTATCATTGGCGCCCTGATTCAGTGCTTTGAATGGAAAATGCTCG
GATCTCAGGGCGAGATCCTGGACCATGGCCGCTCACTGATTAGTATGGATGAACGCCCTGGCCTGACGGCCC
CTCGCGCAAATGACCTGATCGGTATACCTGTCGCTCGCCTGAACCCTACCCCTTTTCGACAGATGTAAttgt
agactc*ggatcc*

GhFNS-II

*gaattc*aaaagatctgagtcttgtagttccgctctgtgtgtcgtcgtatttgtcttagttagttggtttatc
tagctttattgctggagaaacatgaggaatgtataATGCCCTATAAACGTAACCAAAATCACCGCCGCTTAC
CGCCGAGCCCGCCCTCCTTTCCAATCATTGGCCATTTACATCACCTTGGACCGCTCATTCATCAGAGTTTTC
ATGCGCTGTCAACCCGATACGGCTCGCTGATCCATCTGCGCCTGGGTAGCGTGCCGTGCGTGGTTGTGAGTA
CTCCTGATTTGGCAAAAGACTTTCTCAAAACGAACGAACTGGCGTTTTCAAGTCGCAAACATAGCCTCGCGA
TTGATCATATCACCTATGGCGTCGCGTTTGCCTTTGCACCTTACGGTACTTATTGGAAATTTATCAAGAAAC
TGTTTACTGTGGAACTTCTGGGTACGCAGAACCTGAGCCATTTCCTCCCGATCCGGACGCACGAAATTCGTG
AATTGCTTCGCACCCTGATGGTGAAAAGTCGCGCTAAAGAACGTGTGAACCTGACGGAAGAACTCCTGAAAC
TGACTAACAATGTAATCAGCCAGATGATGATGTCTATACGCTGCTCGGGTACAAACTCTGAAGCTGACGAAG
CCAAAAACCTGGTCCGTGAAGTGACCAAGATTTTTGGCCAATTTAACGTGTCTGACTTTATTTGGTTTTGCA
AGAACATTGACCTCCAGGGTTTTAAGAAACGTTATGAGGGCACACATCGCCGTTATGATGCTCTGCTCGAAC
GTATCATTATGGGTCGCGAGGAGAATCGCCGACGCGGAAAAATCAAAGATGGCGAAGGCAAAGATTTCCTTG
ATATGCTGCTGGACGTGCTGGAGGACGGCAAAGCGGAGATTAAGATCACCCGCGATCACATTAAGGCCTTGA
TCTTGGATTTTCTGACCGCCGGCACGGACACTACCGCCATTGCGATCGAGTGGGCACTGGTCGAGCTGATTA
ATAATCCAAACGCGCTGGAAAAAGCCCGCCAAGAAATCGACCAGGTAATTGGCGACGAACGTCTCGTCCAAG
AATCAGATACTCCGAACCTGCCGTATATCCAGGCGATTATCAAAGAAGCGCTGCGCTTGCACCCGCCGATTC
CGATGTTGATCCGGAAATCTACCGAAAATGTTATTGTTCAAGGTTACGACATTCCGGCTGGAACCCTTCTGT
TTGTCAACATCTGGAGCATTGGTCGAAATCCCCAATGTTGGGAAACCCCGTTAGAGTTTAAACCACATCGTT
TCCTGGACGGAGGCGATCTCAAGTCTAGTTTAGATATTAAAGGCCACAACTTCCAACTTCTCCCATTTGGCA
CGGGCAGACGTGGCTGTCCGGGTGTTAACCTGGCCATGCGTGAACTCTCGGTTGTTATTGCGAATCTCATTC
AGTGCTTTGACTGGGATGTTGTAGGTGAGCGCCTGCTGAATACTGATGAACGCGCGGGCTTAACCGCCCCAC
GTGCCGTCGACTTTGTATGCGTGCCTCTGGAACGTGGCAACACGTTAAAAATTCTCGGCTCTAATTAAttgt
agactc*ggatcc*

LjFNS-II

*gaattc*aaaagatctgagtcttgtagtgtccatttctgctttgtaagctaccttgggattgctcactgctga
caaacgggatgaacgatccatacaatcttgcaaggtggtcatccATGGTATTTAAAAAGAATCATAAGCTGC
GTCCTCCGCCGTCTCCTTTCACCCTGCCTATAATTGGGCATCTGCATCTCTTAGGCCCGCTGATTCACCAGA
GTTTCCATCGGTTGAGTACGCTGTATGGCCCATTAATTCAGCTGAAAATCGGATATATCCCTTGTGTAGTAG
CGTCTACCCCGGAACTGGCCAAAGAATTTTTGAAAACCCACGAACTTGCATTTAGCAGCCGCAAACATTCCG
CAGCCATTAAATTGTTGACCTATGATGTGTCATTCGCATTTTCCCCTTATGGCCCTTATTGGAAATTTATTA
AAAAGACCTGTACCTTTGAATTGCTGGGTACACGTAATATGAACCACTTTCTGCCGATCCGTACCAATGAAA
TCCGTCGCTTCTTGCAGGTGATGTTAGAGAAAGCGAAAGCATCCGAAGGTGTTAACGTTACCGAAGAACTGA
TCAAATTAACCAATAATGTTATCAGCCAGATGATGTTCTCTACCCGCTCATCCGGCACGGAAGGCGAAGCGG
AGGAGATGCGCACACTTGTCCGCGAAGTGACACAGATTTTTGGCGAATTTAACGTGTCAGATTTTATTAAGC
TGTGTAAAAACATCGATATTGGCGGCTTCAAAAAGCGCTCGAAAGACATACAGAAACGCTACGACGCGTTGT

TAGAGAAAATTATCTCCGAACGCGAATCAGAACGGGCCCGTAGGGGGAAAAACCGCGAAACACTGGGTGAAG
AAGGCGGAAAAGATTTCCTCGATATGATGCTAGACACCATGGAAGATGGTAAGTGCGAAGTCGAAATCACGC
GAGATCACATCAAGGCATTGGTGTTGGATTTCCTCACCGCCGCGACCGACACCACCGCCATTGCGGTCGAAT
GGACCTTAGCCGAATTAATTTCTAATCCTGAAGTGTTTGATAAGGCACGGGAGGAGATTGATAAAGTGGTTG
GCAAACACCGCCTGGTTACCGAATTAGATACACCGAATTTACCGTACATCCATGCGATCATTAAGGAATCTT
TTCGTCTGCACCCGCCGATTCCGCTTCTTATCCGTAAATCAGTGCAGGATTGCACCGTTGGCGGTTATCACA
TTTCCGCTAACACGATACTGTTCGTTAACATTTGGGCCATCGGCCGCAATCCTAAATATTGGGAATCGCCCA
TGAAATTCTGGCCAGAGCGCTTTTTGGAAAGTAATGGTCCAGGCCCAGTGGGCAGTATGGATATCAAAGGCC
ATCATTATGAACTTTTACCTTTTGGCAGCGGCCGTCGTGGCTGCCCTGGCATGGCGCTTGCGATGCAGGAAC
TTCCGGTGGTTCTGGCGGCGATGATCCAATGCTTTAACTGGAAACCGGTTACGCTCGACGGAGAGGAGCTTG
ACATGTCGGAACGCCCGGGTCTTACGGCGCCCCGTGCACATGATCTCGTGTGCGTGCCTAGCGCCAGGATAA
ATTCCTTTGATAATTTTTAAttgtagactc*ggatcc*

Supplementary Table S3: Plasmids constructed in this study.

Plasmid SBC ID	Plasmid backbone	Genetic construct
SBC015646	p15A	[P_{trc}—RBS—PcFNS-I—T]
SBC015647	pSC101	[P_{trc}—RBS—PcFNS-I—T]
SBC015648	p15A	[P_{lacUV5}—RBS—PcFNS-I—T]
SBC015649	pSC101	[P_{lacUV5}—RBS—PcFNS-I—T]
SBC015650	p15A	[P_{trc}—RBS—PaFNS-I—T]
SBC015651	pSC101	[P_{trc}—RBS—PaFNS-I—T]
SBC015652	p15A	[P_{lacUV5}—RBS—PaFNS-I—T]
SBC015653	pSC101	[P_{lacUV5}—RBS—PaFNS-I—T]
SBC015673	p15A	[P_{trc}—RBS—SbFNS-II—RBS—AtCPR—T]
SBC015674	p15A	[P_{trc}—RBS—ObFNS-II—RBS—AtCPR—T]
SBC015675	p15A	[P_{trc}—RBS—GmFNS-II—RBS—AtCPR—T]
SBC015676	p15A	[P_{trc}—RBS—GhFNS-II—RBS—AtCPR—T]
SBC015677	p15A	[P_{trc}—RBS—LjFNS-II—RBS—AtCPR—T]
SBC015883	p15A	[P_{trc}—RBS—GmF3H—RBS—CuFLS—T]
SBC015884	p15A	[P_{trc}—RBS—GmF3H—T]—[P_{trc}—RBS—CuFLS—T]
SBC015885	p15A	[P_{trc}—RBS—GmF3H—T]—[P_{lacUV5}—RBS—CuFLS—T]
SBC015886	p15A	[P_{lacUV5}—RBS—GmF3H—RBS—CuFLS—T]
SBC015887	p15A	[P_{lacUV5}—RBS—GmF3H—T]—[P_{trc}—RBS—CuFLS—T]
SBC015888	p15A	[P_{lacUV5}—RBS—GmF3H—T]—[P_{lacUV5}—RBS—CuFLS—T]
SBC015889	p15A	[P_{trc}—RBS—CuFLS—RBS—GmF3H—T]
SBC015890	p15A	[P_{trc}—RBS—CuFLS—T]—[P_{trc}—RBS—GmF3H—T]
SBC015891	p15A	[P_{trc}—RBS—CuFLS—T]—[P_{lacUV5}—RBS—GmF3H—T]
SBC015892	p15A	[P_{lacUV5}—RBS—CuFLS—RBS—GmF3H—T]
SBC015893	p15A	[P_{lacUV5}—RBS—CuFLS—T]—[P_{trc}—RBS—GmF3H—T]
SBC015894	p15A	[P_{lacUV5}—RBSCuFLS—T]—[P_{lacUV5}—RBS—GmF3H—T]

Supplementary Table S4: MS parameters and sample dilutions for LC-MS/MS analysis.

Compound	Ionization mode	Parent m/z	Daughter m/z	Dwell time [s]	Cone voltage [V]	Collision energy [V]	Further dilution from quenched samples
Apigenin	Positive	271.0209	152.0209	0.015	94	30	2000
Naringenin	Negative	271.0381	151.0249	0.015	64	18	500 or 2000
p-Coumaric acid	Negative	163.0181	119.0673	0.015	4	14	500 or 2000
Chrysin	Positive	255.0260	152.9334	0.015	4	34	500
Pinocembrin	Negative	255.1543	213.1080	0.015	4	20	500
Cinnamic acid	Negative	147.0005	103.0428	0.015	20	10	500
Luteolin	Positive	287.0819	134.9831	0.015	50	30	100
Eriodictyol	Negative	287.0949	151.0526	0.015	2	14	100
Caffeic acid	Negative	179.0719	135.0888	0.015	32	14	500
Kaempferol	Positive	287.0796	152.9927	0.015	84	30	100 or 500
Dihydrokaempferol	Negative	287.0953	259.1015	0.015	22	14	100 or 500
Quercetin	Negative	301.0746	150.9966	0.015	10	22	8
Dihydroquercetin	Negative	303.0902	285.1047	0.015	2	12	8

Supplementary Table S5: LC-program for LC-MS/MS analysis. LCMS.FLAV-1 was used as default, while LCMS.FLAV-2 was used when experiments included luteolin as an expected analyte.

Program	Time [min]	Flowrate [mL min^{-1}]	%A (water with 0.1% formic acid)	%B (acetonitrile with 0.1% formic acid)
LCMS.FLAV-1	0	0.5	60	40
	1.5	0.5	5	95
	1.9	0.5	5	95
	2	0.5	60	40
	3	0.5	60	40
LCMS.FLAV-2	0	0.5	60.	40
	0.8	0.5	60	40
	1.5	0.5	5	95
	1.9	0.5	5	95
	2	0.5	60	40
	3	0.5	60	40

Supplementary References

Dunstan, M. S., Robinson, C. J., Jervis, A. J., Yan, C., Carbonell, P., Hollywood, K. A., et al. (2020). Engineering *Escherichia coli* towards *de novo* production of gatekeeper (2*S*)-flavanones: naringenin, pinocembrin, eriodictyol and homoeriodictyol. *Synth. Biol.* 5, ysaa012. doi:10.1093/synbio/ysaa012
